# Prevalence and proportion by age and sex of chronic health conditions in a large healthcare system

**DOI:** 10.1371/journal.pone.0308031

**Published:** 2024-09-26

**Authors:** Naomi Gronich, Walid Saliba, Janice B. Schwartz

**Affiliations:** 1 Department of Community Medicine and Epidemiology, Lady Davis Carmel Medical Center, Clalit Health Services, Haifa, Israel; 2 Rappaport Faculty of Medicine, Technion - Israel Institute of Technology, Haifa, Israel; 3 Translational Epidemiology Unit and Research Authority, Lady Davis Carmel Medical Center, Haifa, Israel; 4 Divisions of Geriatrics and Clinical Pharmacology, Department of Medicine, University of California San Francisco, San Francisco, California, United States of America; BSMMU: Bangabandhu Sheikh Mujib Medical University, BANGLADESH

## Abstract

**Background:**

Disease prevalence and distribution by patient characteristics data are needed to guide “representative” patient enrollment in clinical trials and assess relevance of results to patient populations. Our objective was to describe disease prevalence, and age/sex distribution of patients with common chronic conditions from a large population sample.

**Methods:**

A cross-sectional study of all members of Clalit Health Services, alive on January 1, 2020. Included were 26 chronic diseases, and 21 types of malignancies regarded as active by being diagnosed between January 1, 2018- to January 1, 2020, or by prescription of oncologic treatment medications January 1, 2018 and January 1, 2020.

**Results:**

Data from 4,627,183 individuals, 2,274,349 males and 2,352,834 females from newborn to 110 years. Obesity (19%), hypertension (13%), diabetes mellitus (9%), esophagitis-gastritis (5.5%), thyroid disease (5.3%), asthma (5.1%), ischemic heart disease (4.5%), depression (4.5%), osteoporosis (3.8%), and atopic dermatitis (3.6%) were the ten most prevalent conditions. Proportions of age groups varied between conditions (67% of hypertensives were ≥65 years old, 24% ≥80 years; 73% with ischemic heart disease were ≥65 years, 29% ≥80 years; 59% of diabetics were ≥65 years, 17% ≥80 years; 42% of atrial fibrillation patients were ≥80 years; 40% of heart failure patients were ≥80 years). Proportions of males and females for most conditions paralleled prevalence except proportions of women increased after age 80 for cardiovascular diseases, and for diabetes after age 75. The five most frequent active cancers were breast, prostate, colon/rectal, lymphoma and melanoma. The prevalence of cancers increased with age beginning in the middle-aged groups and peaking at very old ages. Women had lower prevalence of lung cancers and accounted for lower percentages of patients with lung cancers (45 vs 55%) but similar percentages for women and men were seen in the patients with colon and rectal cancer (50.4 vs. 49.6% in women) and lymphoma (50.7 vs. 49.3% in women).

**Conclusions:**

Prevalence of medical conditions and distributions differ by age and sex. This information serves as an example and resource for data needed to describe a “representative” clinical population.

## 1. Introduction

Enrollment of participants into clinical trials of new products should represent the population the product in intended for [1–6]. Nevertheless, under-representation of certain subgroups of patients in clinical trials has been recently noted, and recommendations to address this situation have been made [4, 7–9].

A challenge has been how to define representativeness. An emerging consensus is that this should be based on the epidemiology of the disease. In other words, that participants in a trial should approximate their presence in the clinical population and not their proportion of the overall population [10–12]. However, in many instances, these data are not readily available.

This principle has been cited in recent U.S. legislation on clinical trial diversity and modernization [13]. The legislation requires that sponsors submit a study enrollment plan and provide the rationale for the plan that may be based on a group’s “estimated prevalence or incidence in the United States of the disease or condition for which the drug or device is being investigated in the relevant clinical trial, if such estimated prevalence or incidence is known or can be determined based on available data [13].

Population data on age, sex, and racial characteristics are routinely collected by the U.S. census bureau but granular data on health condition prevalence by age, sex, and racial subgroups are not as regularly assessed, collected, reported, or made publicly available. These data are essential to meeting the goal of having clinical trial populations mirror patient populations and are key to the accountability of enrollment in trials. Such data exist in databases of countries with single healthcare provision systems and in large healthcare system databases.

Our goal was to present data on the prevalence of common chronic conditions from a clinical registry of a large non-profit health maintenance organization and to provide the proportions of patients with the conditions by age and sex. Our intent was that this information serve as an example and resource for data needed to describe a “representative” clinical population.

## 2. Methods

This is a retrospective cross-sectional study conducted within the Clalit Chronic Diseases Registry of Clalit Health Services (CHS) in Israel. Health care coverage in Israel is mandatory according to the National Health Insurance Law (1995) and is provided by four groups akin to not-for-profit health maintenance organizations. CHS is one of the four groups and provides inclusive health care for more than half of the Israeli population (~4.6 million—2,274,349 males and 2,352,834 females, as of January 1, 2020, mostly white population with 98% White, 2% Ethiopian origin). CHS maintains a database that receives data from multiple sources including records of primary care physicians, community specialty clinics, hospitalizations, laboratories, pharmacies, and cancer diagnosis (ICD-O-3) from the Israeli National Cancer Registry (INCR). A registry of chronic diseases diagnoses is compiled from these data sources. Diagnoses are captured in the registry by diagnosis-specific algorithms, employing International Classification of Diseases Ninth revision (ICD-9) and ICD-O-3 code reading, laboratory test results and disease-specific drug usage. A record is kept of the data-sources and dates used to establish the diagnosis, with the earliest recorded date, from any source, considered to be the defining date of diagnosis. Designed for purposes of administrative and clinical management, the database is available for clinical studies. The validity of selected disease diagnoses in the CHS database was found to be high in previous studies [14–17]. The study was approved by the institutional review board of Lady Davis Medical Center (CMC-0039-23) and Data Utilization Committee of CHS. Owing to the retrospective nature of the study, a waiver of informed consent was granted by the institutional reviewed board.

This cross-sectional study consisted of all CHS members up to 110 years of age who were alive on January 1, 2020. There were no exclusion criteria. Twenty-six major chronic diseases, including obesity, were included (Table 1). In addition, 21 types of malignancies were included, regarded as active by being diagnosed any time between January 1, 2018- to January 1, 2020, or by prescription of oncologic treatment medications (ATC codes L01, L02) between January 1, 2018 and January 1, 2020.

**Table 1:** Prevalence of medical conditions in a large healthcare system.

Condition*	Total Number of Patients (4,627,183)	Prevalence (%)
Asthma	233,453	5.1
Atopic dermatitis	166,961	3.6
Atrial fibrillation	86,085	1.9
Cerebrovascular accident/transient ischemic attack	120,475	2.6
Chronic obstructive pulmonary disease	84,087	1.8
Congestive heart failure	54,871	1.2
Dementia	52,165	1.1
Depression (including bipolar disease)	208,947	4.5
Diabetes mellitus	400,319	8.7
Dialysis (chronic)	6,364	0.1
Epilepsy	49,422	1.1
Gastroesophageal reflux, gastritis, duodenitis	254,094	5.5
Glaucoma	84,201	1.8
Gout	27,890	0.6
Hypertension	597,762	12.9
Ischemic heart disease	209,026	4.5
Obesity	859,945	18.6
Osteoarthritis	100,113	2.2
Osteoporosis	175,924	3.8
Parkinson’s disease	17,830	0.4
Peripheral artery disease (including aortic aneurysm)	79,733	1.7
Prostatic hypertrophy, benign	89,702	1.9
Renal failure, chronic	84,177	1.8
Rheumatoid arthritis	22,362	0.5
Schizophrenia	33,087	0.7
Thyroid disease	245,676	5.3
Malignant diseases		1.41
Bladder	3,297	0.07
Bone	413	0.01
Brain / CNS	961	0.02
Breast	21,180	0.46
Colon or rectum	5,442	0.12
Connective tissue / sarcoma	1,103	0.02
Esophagus	266	0.01
Kidney	2,094	0.05
Larynx	533	0.01
Liver / bile ducts	573	0.01
Lung	3,441	0.07
Lymphoma	4,578	0.10
Melanoma	3,772	0.08
Multiple myeloma	1,566	0.03
Ovary	1,034	0.02
Pancreas	864	0.02
Pharynx	815	0.02
Prostate	6,870	0.15
Stomach	975	0.02
Thyroid	1,826	0.04
Uterus, cervix	3603	0.08

*By alphabetical order

The prevalence of each medical condition is reported by age within 22 groups of five-year intervals from birth to 110 years with an additional stratification by sex. The prevalence of each condition within each age/sex group was calculated by dividing the number of prevalent cases within the group by the total number of individuals within that group, as of January 1, 2020. In addition, we present the distribution of age of patients for each medical condition. The proportion of each age group was calculated by dividing the number of prevalent cases in the age group by the total number of prevalent cases, as of January 1, 2020. Each analysis is provided for the total population and for men and women. Data was accessed on August 2, 2023. Authors had no access to information that could identify individual participants for this study, during or after data collection.

## 3. Results

There were 4,627,183 people insured by CHS, including 2,274,349 males and 2,352,834 females as of January 1, 2020. The overall prevalence of 26 chronic diseases and 21 active malignancies within the CHS population is presented in Table 1.

Obesity (19%), hypertension (13%), and diabetes mellitus (9%), followed by reflux esophagitis-gastritis-duodenitis (5.5%), thyroid disease (5.3%), asthma (5.1%), ischemic heart disease (4.5%), depression (including manic depressive disorder, 4.5%), osteoporosis (3.8%), and atopic dermatitis (3.6%) were the ten most prevalent chronic medical conditions considering all age groups. A few of these are discussed below and data for the other conditions are presented in full in the S1 File.

**Fig 1:**
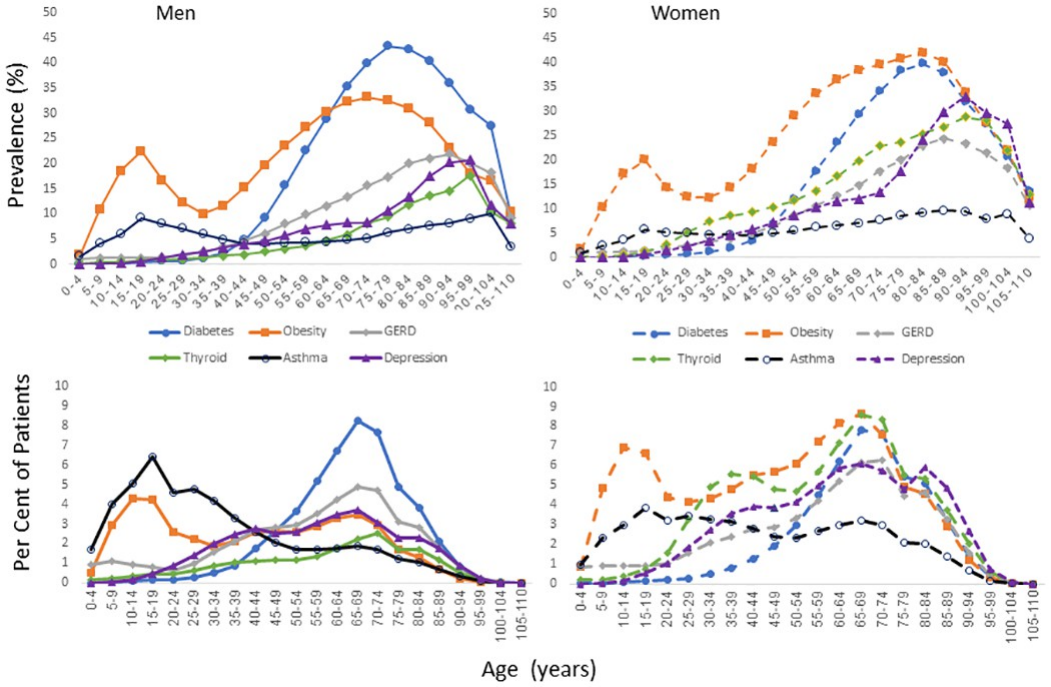
Prevalence and proportion of patients by age and sex for the most common non-cardiovascular chronic conditions in adults: Obesity, diabetes, gastroesophageal reflux disease (GERD), thyroid diseases, asthma, and depression.

Obesity (see Table 2 and Fig 1). Age-related trends were seen with obesity present in about 11% at ages 5–9 years and peaking in the 15–19 teen years, then steadily increasing again after ages 35–39, and greater in women than men (after age 30 years). Twenty-seven percent of obese patients were age 65 and above while only 7 percent were age 80 and above.

**Table 2:** Age and sex distribution of enrollees for the three most common health conditions.

**a: Obesity**									
				**Obesity**					
	Clalit Enrollees (No.)			**Prevalence (%)**			**Per cent of Obese**		
**Age (y)**	Male(M)	Female(F)	Total	M	F	All	M	F	All
0–4	248558	234757	483315	1.8	1.7	1.8	0.5	0.5	1.0
5–9	231281	220106	451387	10.9	10.4	10.7	2.9	2.7	5.6
10–14	199945	190186	390131	18.6	17.2	17.9	4.3	3.8	8.1
15–19	163169	156268	319437	22.4	20.0	21.2	4.3	3.6	7.9
20–24	132509	143680	276189	16.6	14.4	15.5	2.6	2.4	5.0
25–29	156927	159023	315950	12.3	12.4	12.3	2.2	2.3	4.5
30–34	162004	165350	327354	10.0	12.3	11.2	1.9	2.4	4.3
35–39	156661	158737	315398	11.6	14.3	13.0	2.1	2.6	4.8
40–44	145144	143572	288716	15.3	18.1	16.7	2.6	3.0	5.6
45–49	115926	112939	228865	19.7	23.7	21.7	2.7	3.1	5.8
50–54	93815	98655	192470	23.6	29.0	26.4	2.6	3.3	5.9
55–59	91223	101690	192913	27.2	33.5	30.5	2.9	4.0	6.8
60–64	93150	105570	198720	30.4	36.4	33.6	3.3	4.5	7.8
65–69	93093	106207	199300	32.3	38.5	35.6	3.5	4.7	8.2
70–74	76573	90083	166656	33.2	39.5	36.6	3.0	4.1	7.1
75–79	45011	56829	101840	32.4	40.9	37.1	1.7	2.7	4.4
80–84	36050	51508	87558	31.0	42.0	37.4	1.3	2.5	3.8
85–89	21131	34197	55328	28.1	40.1	35.5	0.7	1.6	2.3
90–94	9147	17225	26372	23.1	33.8	30.1	0.2	0.7	0.9
95–99	2571	5410	7981	18.1	27.6	24.5	0.1	0.2	0.2
100–104	374	665	1039	16.6	22.0	20.0	0.0	0.0	0.0
105–110	87	177	264	10.3	11.9	11.4	0.0	0.0	0.0
Total	2274349	2352834	4627183	17.1	20.0	18.6	45.2	54.8	
**b: Hypertension**									
				**Hypertension**					
	Clalit Enrollees (No.)			**Prevalence (%)**			**Per cent of Hypertensives**		
**Age (y)**	Male(M)	Female(F)	Total	M	F	All	M	F	All
0–4	248558	234757	483315	0.0	0.0	0.0	0.0	0.0	0.0
5–9	231281	220106	451387	0.0	0.0	0.0	0.0	0.0	0.0
10–14	199945	190186	390131	0.1	0.1	0.1	0.0	0.0	0.0
15–19	163169	156268	319437	0.2	0.1	0.2	0.1	0.0	0.1
20–24	132509	143680	276189	0.5	0.2	0.4	0.1	0.1	0.2
25–29	156927	159023	315950	0.8	0.4	0.6	0.2	0.1	0.3
30–34	162004	165350	327354	1.3	0.8	1.0	0.4	0.2	0.6
35–39	156661	158737	315398	2.4	1.5	2.0	0.6	0.4	1.0
40–44	145144	143572	288716	5.0	3.6	4.3	1.2	0.9	2.1
45–49	115926	112939	228865	10.1	8.0	9.1	2.0	1.5	3.5
50–54	93815	98655	192470	17.6	14.8	16.1	2.8	2.4	5.2
55–59	91223	101690	192913	27.7	23.2	25.3	4.2	3.9	8.2
60–64	93150	105570	198720	38.3	32.8	35.4	6.0	5.8	11.8
65–69	93093	106207	199300	50.2	43.7	46.7	7.8	7.8	15.6
70–74	76573	90083	166656	61.2	57.0	58.9	7.8	8.6	16.4
75–79	45011	56829	101840	68.1	66.8	67.4	5.1	6.3	11.5
80–84	36050	51508	87558	75.0	76.5	75.9	4.5	6.6	11.1
85–89	21131	34197	55328	79.8	82.0	81.2	2.8	4.7	7.5
90–94	9147	17225	26372	81.3	85.5	84.0	1.2	2.5	3.7
95–99	2571	5410	7981	80.9	85.0	83.7	0.3	0.8	1.1
100–104	374	665	1039	67.4	77.6	73.9	0.0	0.1	0.1
105–110	87	177	264	40.2	48.0	45.5	0.0	0.0	0.0
Total	2274349	2352834	4627183	12	13	13	47.3	52.7	
**c: Diabetes Mellitus**									
				**Diabetes Mellitus**					
	Clalit Enrollees (No.)			**Prevalence (%)**			**Per cent of Diabetics**		
**Age (y)**	Male(M)	Female(F)	Total	M	F	All	M	F	All
0–4	248558	234757	483315	0.0	0.0	0.0	0.0	0.0	0.0
5–9	231281	220106	451387	0.1	0.1	0.1	0.0	0.0	0.1
10–14	199945	190186	390131	0.2	0.2	0.2	0.1	0.1	0.2
15–19	163169	156268	319437	0.5	0.4	0.4	0.2	0.2	0.4
20–24	132509	143680	276189	0.6	0.6	0.6	0.2	0.2	0.4
25–29	156927	159023	315950	0.7	0.7	0.7	0.3	0.3	0.6
30–34	162004	165350	327354	1.2	1.2	1.2	0.5	0.5	1.0
35–39	156661	158737	315398	2.3	2.0	2.1	0.9	0.8	1.7
40–44	145144	143572	288716	4.8	3.5	4.1	1.7	1.3	3.0
45–49	115926	112939	228865	9.1	6.8	8.0	2.6	1.9	4.6
50–54	93815	98655	192470	15.6	12.1	13.8	3.7	3.0	6.6
55–59	91223	101690	192913	22.7	17.8	20.1	5.2	4.5	9.7
60–64	93150	105570	198720	28.9	23.6	26.1	6.7	6.2	13.0
65–69	93093	106207	199300	35.4	29.4	32.2	8.2	7.8	16.0
70–74	76573	90083	166656	40.0	34.0	36.7	7.6	7.7	15.3
75–79	45011	56829	101840	43.4	38.3	40.6	4.9	5.4	10.3
80–84	36050	51508	87558	42.7	39.8	41.0	3.8	5.1	9.0
85–89	21131	34197	55328	40.5	37.9	38.9	2.1	3.2	5.4
90–94	9147	17225	26372	35.9	32.0	33.4	0.8	1.4	2.2
95–99	2571	5410	7981	30.8	27.4	28.5	0.2	0.4	0.6
100–104	374	665	1039	27.5	20.9	23.3	0.0	0.0	0.1
105–110	87	177	264	9.2	13.6	12.1	0.0	0.0	0.0
Total	2274349	2352834	4627183	8.8	8.5	8.7	50	50	

**Fig 2:**
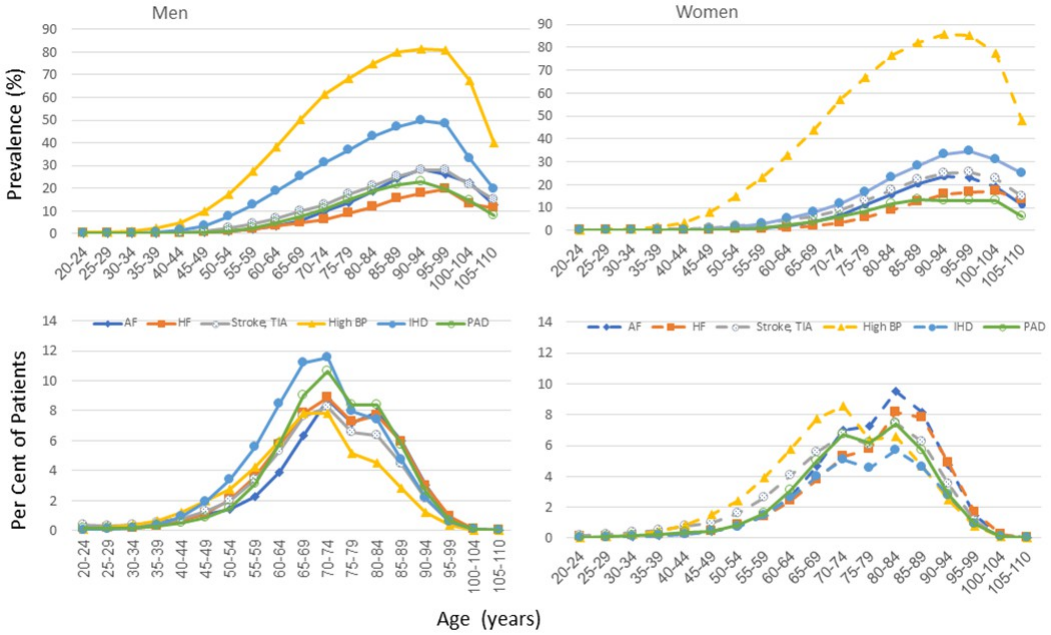
Prevalence and proportion of patients by age and sex for common cardiovascular conditions in adults: High blood pressure (BP), ischemic heart disease (IHD), stroke/transient ischemic attack (TIA), atrial fibrillation (AF), peripheral artery disease (PAD), heart failure (HF).

Hypertension (see Table 2, and Fig 2). The prevalence of hypertension steadily increased throughout the adult years reaching 35% at ages 60–64 and over 75% in those age 80 years and older. Adults age 65 and above comprised greater proportions of the hypertensive patients with 67% age 65 or older, and 24% age 80 or older. There were more men with hypertension than women until age 65; and more women than men after age 70.

Diabetes (see Table 2 and Fig 1). The prevalence of diabetes reached 20% at ages 55–59 years and peaked at 41% at ages 75–84 years. Adults age 65 and above accounted for 59% of the diabetic population with 17% over the age of 80 years. Prevalence was higher in men than women across the adult age span, however, women were a higher proportion of diabetics than men after age 75.

Ischemic Heart Disease (IHD) (see Fig 2 and e-Table 15b in S1 File). The prevalence of IHD reached 12% at ages 60–64, 26% at ages 75–79, and peaked at 39% at ages 90–99 years (see Fig 2). Seventy-three percent of IHD patients were age 65 or older, 42% were age 75 or older, and 29% were age 80 years or older. Prevalence was higher in men at all ages with men accounting for two-thirds of patients with ischemic heart disease. See Fig 2 and e-Tables for data for atrial fibrillation (e-Table 3 in S1 File), congestive heart failure (e-Table 6 in S1 File), cerebrovascular disease (stroke and transient ischemic attack e-Table 4 in S1 File), and peripheral vascular disease (e-Table 21 in S1 File).

Asthma (Fig 1 and e-Table 1 in S1 File). The prevalence of asthma increased in childhood to a peak of 7.5% in the teen years between 15–19 years, and then decreased in adulthood and rose slightly at older ages from 60 years of age onward. Thirty five percent of asthma patients were less than 25 years of age, 20% were age 65 or older, 10% were age 75 or older, and 6.5% were age 80 or older. The percent of male and female patients with asthma overall was about equal (51% male, 49% female).

Depression. (Fig 1 and e-Table 8 in S1 File). The prevalence of depression increased to 10% at age 65 and was documented in about a quarter of adults aged 85 and above. Forty-seven percent of patients with depression were age 65 and older, 28% age 75 and older, and 20% were age 80 years and older. From age 15 years upward, there were about twice as many women than men with depression with females accounting for 64% of all patients with depression.

**Fig 3:**
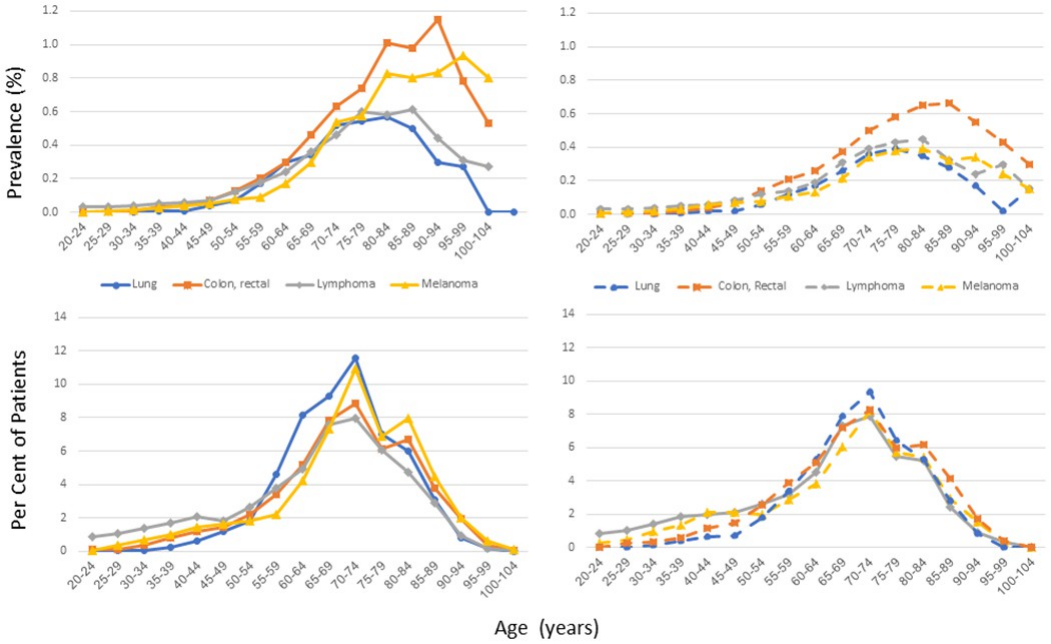
Prevalence and proportion of patients by age and sex for the most common cancers in both adult sexes: Colorectal, lymphoma, melanoma, and lung cancer. Prostate cancer within men, and breast cancer within women are presented in e-Figs 2 and 3 in S1 File.

Cancers. The five most frequent active cancers were breast, prostate, colon/rectal, lymphoma and melanoma (Table 1). Prevalence and proportion of patients with the cancer by age and sex are shown in Fig 3 for the five most frequent cancers in both men and women and in Supplemental figures and Tables for the remainder listed in Table 1. The prevalence of cancers increased with age beginning in the middle-aged groups and peaking at very old ages. Women had lower prevalence of lung cancers and accounted for lower percentages of patients with lung cancers (45 vs 55%) but similar percentages for women and men were seen in the patients with colon and rectal cancer (50.4 vs. 49.6% in women) and lymphoma (50.7 vs. 49.3% in women).

Data on prevalence and proportions of patients by age and sex for the remainder of the conditions listed in Table 1 are in the on-line e-Tables. Conditions with age trends included atopic dermatitis being more frequent in children and teenagers, schizophrenia peaking in middle age, and higher prevalence of chronic obstructive pulmonary disease, glaucoma, and Parkinson’s disease at older ages. Dementia, osteoarthritis, osteoporosis prevalence was greater at older ages and more prevalent in women accounting for two-thirds of dementia patients, three quarters of osteoarthritis patients, and 88 percent of osteoporosis patients. Rheumatoid arthritis has higher prevalence in females than men and three quarters of patients with rheumatoid arthritis were women. Sex differences were also seen for renal disease with a higher prevalence in men and greater proportions of chronic renal disease and dialysis patients being men. In addition, gout prevalence was fourfold greater in men than in women, and men comprised 83% of gout patients.

## 4. Discussion

A number of groups have been under-represented in clinical trials of new therapies with older adults and women being among those under-represented [9, 10, 18–23]. Older adults are an increasing proportion of the U.S. population and the major consumers of prescription medications [24, 25]. Yet, older adults have been under-represented in the evaluation of medications resulting in a dearth of information to optimize use of drugs in this population [10, 11, 19]. With increasing emphasis and legal imperatives to enroll of “representative” patient groups in clinical trials, data are needed to define a “representative” sample. Data are needed on population demographics and disease prevalence in order to identify proportions of patient populations by characteristics such as age, sex, race, as well as social determinants of health. In this article, we present medical condition prevalence data by age subgroups of common medical conditions and active malignancies in 4.75 million people enrolled in the Clalit Health Services (CHS) that provides comprehensive care for slightly more than 50% of Israeli residents. We also provide information on the proportion of the clinical patients with each condition by age and by sex.

Our results show the impact of a changing demographic profile with growing numbers of older adults and more women than men at older ages on the composition of patient populations with common medical conditions. A few examples of age-related and sex-related findings are discussed below. Both the prevalence of hypertension and the proportion of patients with hypertension were high for older adults, yet perhaps surprising is that two-thirds of hypertensive patients were over the age of 65 years, one third were over the age of 75, and about a quarter of patients with hypertension were over 80 years of age. While expected that one-third of patients with strokes were over the age of 80 years, it is important to note that 42% of atrial fibrillation patients were over age 80, and 40% of heart failure patients were over age 80. The proportions of patients over 80 and women being cared for with these conditions are in stark contrast to the age and sex enrollment in pivotal clinical trials for these conditions [10, 21, 26]. Similarly, malignancies were diseases appearing in middle-age and peaking at older ages that have been under-represented in clinical trials [27, 28].

It is key to the potential relevance of our findings that similar prevalence in older adults for hypertension, ischemic heart disease and osteoporosis were reported in a profile of seniors in Canada from the same time point [29], in a slightly earlier survey of about a million older adults in Italy [30], and in a U.S. Profile of Older Americans [24]. Israel, like the U.S., is categorized as a very high Human Development Index (HDI) country [31, 32] and age-standardized incidence of many chronic diseases, including cancer, are expected to be similar [33–37]. For example, age-standardized prevalence of raised blood pressure in adults aged 18 years and over in 2014 was very similar in USA and Israel [38]. Osteoarthritis, however, was reported at higher prevalence in older adults in Canada (38%) and in the U.S than in the CHS data (12%), suggesting under-reporting of osteoarthritis in Israel.

Children accounted for 35% of asthma patients as asthma is recognized as the most common chronic disease in children. Inhalable steroids are approved for use in children and the trial of the only combination agent currently approved for use in children included adolescent, albeit at lower proportions than percentages adolescents in the asthma population [39]. Similarly, with the increase in obesity that now includes children who comprised 23% of the obese patient population, smaller clinical trials of newer agents in adolescents have been conducted but not with numbers proportional to the adolescent portion of the obese population. Children accounted for 60% of atopic dermatitis diagnoses. About one third were adolescents that either have not been enrolled or enrolled in relatively small numbers in recent trials of agents for moderate to severe atopic dermatitis [40–42].

Sex-related differences in disease prevalence included the higher prevalence of thyroid disease and depression in women compared to men at all ages. In contrast, while diabetes was more prevalent in men than women across the adult age span, women comprised a higher proportion of patients with diabetes than men after age 75. Women also had higher prevalence of hypertension than men after age 70. Similarly, while men comprised two-thirds of patients with ischemic heart disease, after age 80 half of patients with ischemic heart disease were women.

Previous literature has been mainly reviewing chronic diseases incidence, prevalence, and mortality [43–45]. For example The Global Burden of Diseases, Injuries, and Risk Factors Study (GBD), provides a systematic assessment of published, publicly available, and contributed data on incidence, prevalence, and mortality for an exhaustive list of diseases and injuries [46]. In other instances, age distribution is extrapolated from data on disease prevalence [47]. Only few data sources have been utilized to describe age distribution of all patients with certain diseases in a population [48].

The strengths of the data include the large sample size, that data are reported without arbitrary age cutoffs or age groupings, the comprehensiveness regarding the number of chronic conditions combined in one report, and the use of laboratory and prescription data in classifying diagnoses. However, there are limitations. This single large health care organization lacks racial diversity in the population enrolled reflecting the demographics of the country it serves. CHS also has a greater proportion of enrollees in the pediatric age group than the U.S. while the proportion and age distribution of young and older adults are similar to the U.S. (see e-Fig 1 in S1 File). Diagnoses rely on coded data, laboratory tests performed, and prescriptions ordered and some inaccuracies may have occurred. The data also reflect a snapshot at one recent time point.

## 5. Conclusion

We report comprehensive data on prevalence of chronic disease and proportions of patients within chronic conditions by age and sex from an electronic registry of patients enrolled in a large healthcare system. The data demonstrates differences in the prevalence of many common chronic medical conditions by age and by sex. The data also demonstrate instances where prevalence of a condition within age groups or by sex in a population differ markedly from the proportion of the total disease population that the subgroup comprises. The data provide an example and resource for defining age and sex representative populations for evaluation of therapies for multiple common chronic conditions in similar demographic populations.

## Supporting information

S1 File (PDF)
