# Supplementary material for: Prevalence and proportion by age and sex of chronic health conditions in a large healthcare system
Source: PLoS One. 2024 Sep 26;19(9):e0308031. doi: 10.1371/journal.pone.0308031 (PMC11426542; doi:10.1371/journal.pone.0308031)
Supplement: S1 File — (PDF) [file pone.0308031.s001.pdf]

Supplementary File

Prevalence and Proportion by Age and Sex of Chronic Health Conditions in a Large Healthcare System

Naomi Gronich, MD, Walid Saliba, MD, MPH, and Janice B. Schwartz, MD

e- Figure 1. Comparison of the Clalit Health Services (CHS) patient population (from database) and the U.S. total population (from census data)

e- Figure 2. Prevalence and proportions of prostate cancer in men by age

e- Figure 3. Prevalence and proportions of breast cancer in women by age

On-line e-Tables of Disease Prevalence and Proportions of Patient Population by Age and Sex

E-TABLE 1	Asthma
E-TABLE 2	Atopic dermatitis
E-TABLE 3	Atrial fibrillation
E-TABLE 4	Cerebrovascular accident/transient ischemic attack
E-TABLE 5	Chronic obstructive pulmonary disease
E-TABLE 6	Congestive heart failure
E-TABLE 7	Dementia
E-TABLE 8	Depression (including bipolar disease)
E-TABLE 9	Diabetes mellitus
E-TABLE 10	Dialysis, chronic
E-TABLE 11	Epilepsy
E-TABLE 12	Gastroesophageal reflux, gastritis, duodenitis
E-TABLE 13	Glaucoma
E-TABLE 14	Gout
E-TABLE 15	Hypertension
E-TABLE 16	Ischemic heart disease
E-TABLE 17	Obesity
E-TABLE 18	Osteoarthritis
E-TABLE 19	Osteoporosis
E-TABLE 20	Parkinson's disease
E-TABLE 21	Peripheral vascular disease (including aortic disease)
E-TABLE 22	Prostatic hypertrophy, benign
E-TABLE 23	Renal failure, chronic
E-TABLE 24	Rheumatoid arthritis

E-TABLE 25	Schizophrenia
E-TABLE 26	Thyroid disease
E-TABLE 27	Bladder cancer
E-TABLE 28	Bone cancer
E-TABLE 29	Brain/CNS cancer
E-TABLE 30	Breast cancer
E-TABLE 31	Colon, rectal cancer
E-TABLE 32	Connective tissue, sarcoma cancer
E-TABLE 33	Esophageal cancer
E-TABLE 34	Kidney cancer
E-TABLE 35	Laryngeal cancer
E-TABLE 36	Liver/bile duct cancer
E-TABLE 37	Lung cancer
E-TABLE 38	Lymphoma
E-TABLE 39	Malignant melanoma
E-TABLE 40	Multiple myeloma
E-TABLE 41	Ovarian cancer
E-TABLE 42	Pancreatic cancer
E-TABLE 43	Pharyngeal cancer
E-TABLE 44	Prostate cancer
E-TABLE 45	Stomach cancer
E-TABLE 46	Thyroid cancer
E-TABLE 47	Uterine, cervix cancer

E-Fig 1. Comparison of the Clalit Health Services (CHS) patient population (from database) and the U.S. total population (from census data)

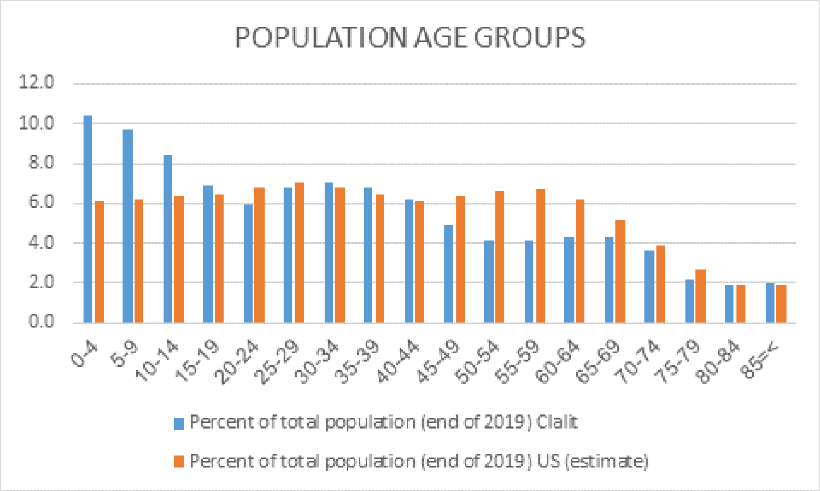

E-Fig 2. Prevalence (upper panel) and proportions (lower panel) of prostate cancer in men by age, CHS

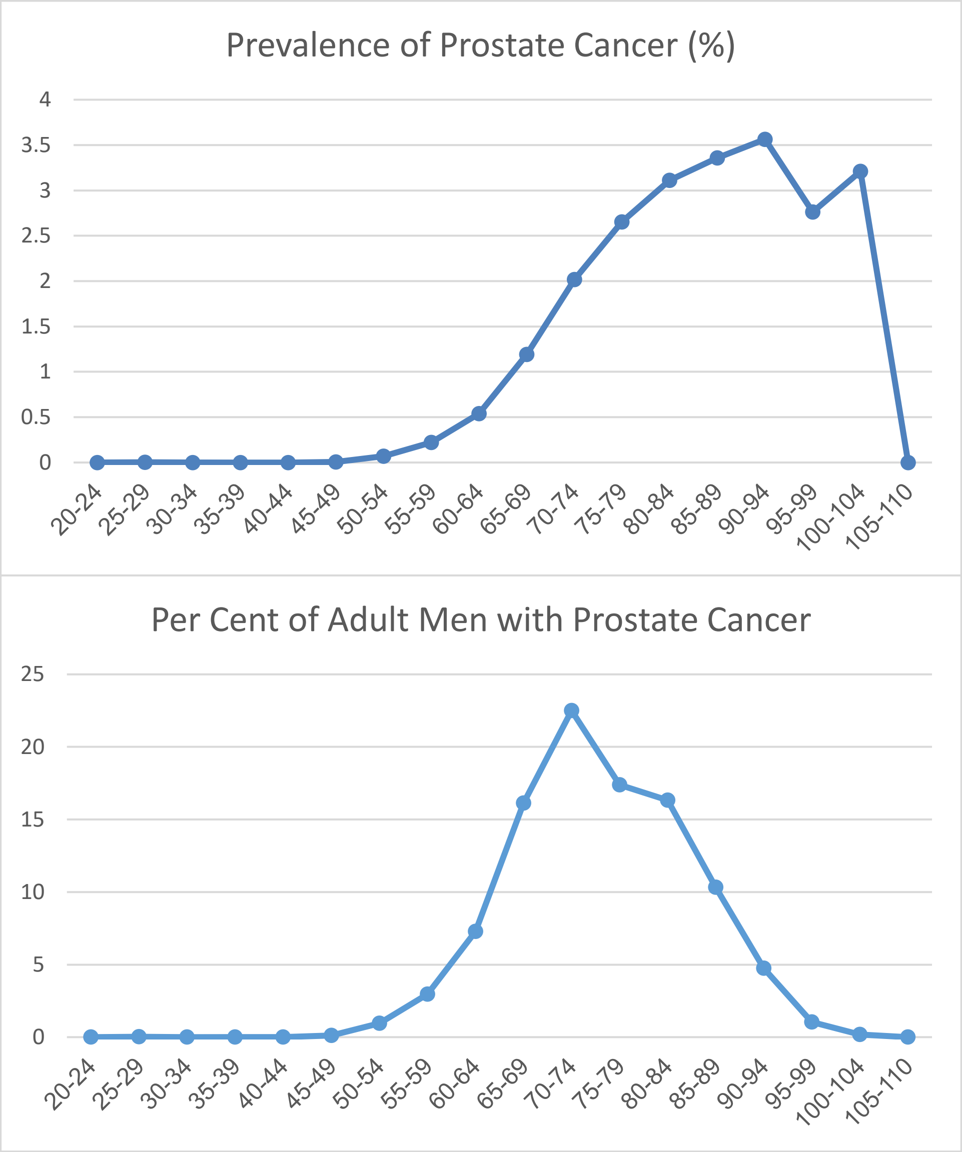

Age (years)

E-Fig 3. Prevalence (upper panel) and proportions (lower panel) of breast cancer in women by age, CHS

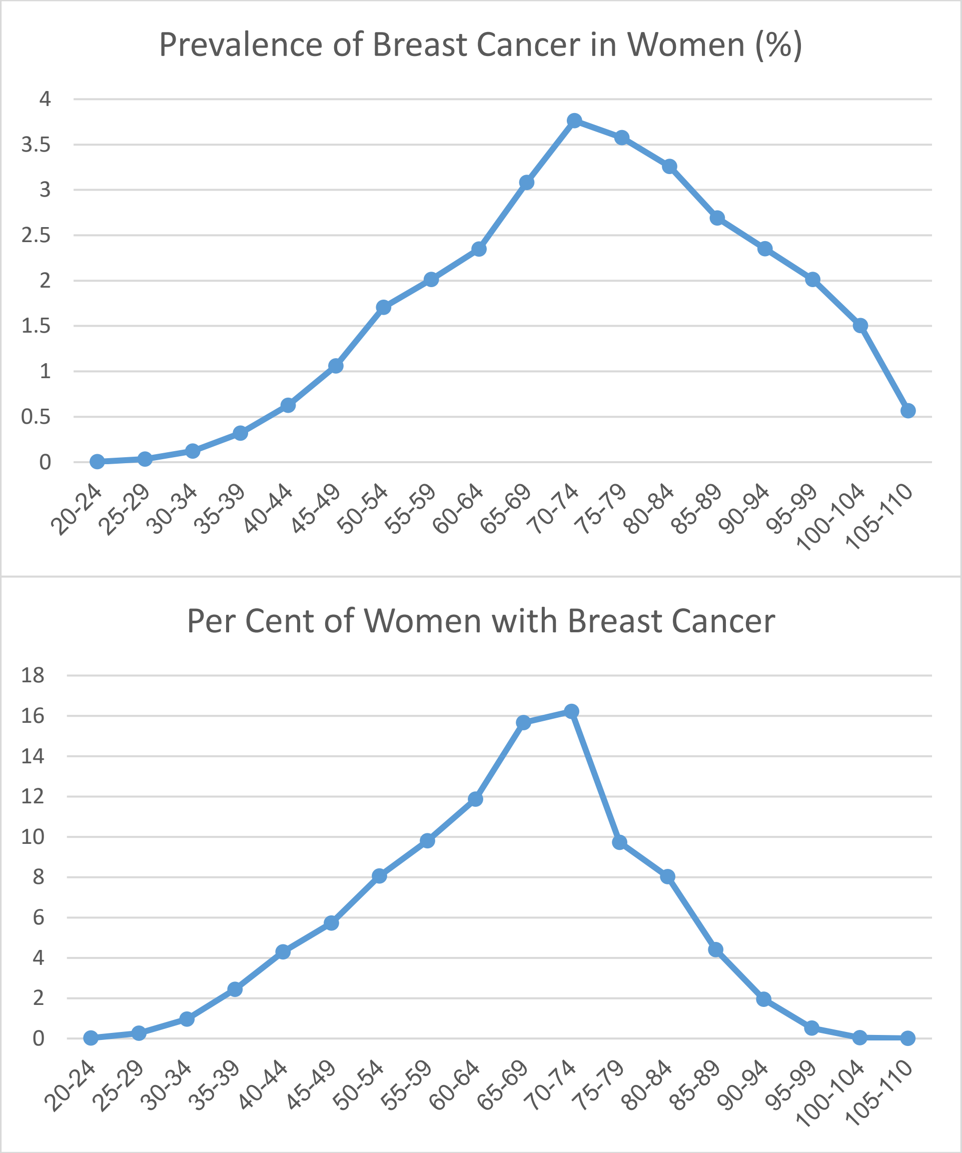

Age (years)

eTable 1. Asthma prevalence and proportions of patient population by age and sex

	Asthma (n)			Percent of asthma patients			Total population (n)			Prevalence (%)		
**Age group**	**Male**	**Female**	**Total**	**Male**	**Female**	**Total**	**Male**	**Female**	**Total**	**Male**	**Female**	**Total**
0-4	3981	2289	6270	1.71	0.98	2.69	248558	234757	**483315**	1.60	0.98	**1.30**
5-9	9402	5468	14870	4.03	2.34	6.37	231281	220106	**451387**	4.07	2.48	**3.29**
10-14	11811	6919	18730	5.06	2.96	8.02	199945	190186	**390131**	5.91	3.64	**4.80**
15-19	14966	9001	23967	6.41	3.86	10.27	163169	156268	**319437**	9.17	5.76	**7.50**
20-24	10663	7465	18128	4.57	3.20	7.77	132509	143680	**276189**	8.05	5.20	**6.56**
25-29	11142	8014	19156	4.77	3.43	8.21	156927	159023	**315950**	7.10	5.04	**6.06**
30-34	9727	7714	17441	4.17	3.30	7.47	162004	165350	**327354**	6.00	4.67	**5.33**
35-39	7692	7437	15129	3.29	3.19	6.48	156661	158737	**315398**	4.91	4.69	**4.80**
40-44	6022	6545	12567	2.58	2.80	5.38	145144	143572	**288716**	4.15	4.56	**4.35**
45-49	4805	5651	10456	2.06	2.42	4.48	115926	112939	**228865**	4.14	5.00	**4.57**
50-54	3973	5483	9456	1.70	2.35	4.05	93815	98655	**192470**	4.23	5.56	**4.91**
55-59	3961	6327	10288	1.70	2.71	4.41	91223	101690	**192913**	4.34	6.22	**5.33**
60-64	4096	7005	11101	1.75	3.00	4.76	93150	105570	**198720**	4.40	6.64	**5.59**
65-69	4415	7487	11902	1.89	3.21	5.10	93093	106207	**199300**	4.74	7.05	**5.97**
70-74	3965	6935	10900	1.70	2.97	4.67	76573	90083	**166656**	5.18	7.70	**6.54**
75-79	2814	4909	7723	1.21	2.10	3.31	45011	56829	**101840**	6.25	8.64	**7.58**
80-84	2524	4770	7294	1.08	2.04	3.12	36050	51508	**87558**	7.00	9.26	**8.33**
85-89	1613	3314	4927	0.69	1.42	2.11	21131	34197	**55328**	7.63	9.69	**8.91**
90-94	745	1636	2381	0.32	0.70	1.02	9147	17225	**26372**	8.14	9.50	**9.03**
95-99	233	426	659	0.10	0.18	0.28	2571	5410	**7981**	9.06	7.87	**8.26**
100-104	38	60	98	0.02	0.03	0.04	374	665	**1039**	10.16	9.02	**9.43**
105-110	3	7	10	0.00	0.00	0.00	87	177	**264**	3.45	3.95	**3.79**
**Total**	**118591**	**114862**	**233453**	**50.80**	**49.20**		**2274349**	**2352834**	**4627183**	**5.21**	**4.88**	**5.05**

eTable 2. Atopic dermatitis prevalence and proportions of patient population by age and sex

	Atopic dermatitic (n)			Percent of atopic dermatitis patients			Total population (n)			Prevalence (%)		
**Age group**	**Male**	**Female**	**Total**	**Male**	**Female**	**Total**	**Male**	**Female**	**Total**	**Male**	**Female**	**Total**
0-4	10393	8223	**18616**	6.22	4.93	11.15	248558	234757	**483315**	4.18	3.50	**3.85**
5-9	16379	14395	**30774**	9.81	8.62	18.43	231281	220106	**451387**	7.08	6.54	**6.82**
10-14	17034	14873	**31907**	10.20	8.91	19.11	199945	190186	**390131**	8.52	7.82	**8.18**
15-19	9791	9662	**19453**	5.86	5.79	11.65	163169	156268	**319437**	6.00	6.18	**6.09**
20-24	3733	5582	**9315**	2.24	3.34	5.58	132509	143680	**276189**	2.82	3.89	**3.37**
25-29	3122	5668	**8790**	1.87	3.39	5.26	156927	159023	**315950**	1.99	3.56	**2.78**
30-34	2775	5586	**8361**	1.66	3.35	5.01	162004	165350	**327354**	1.71	3.38	**2.55**
35-39	2645	5433	**8078**	1.58	3.25	4.84	156661	158737	**315398**	1.69	3.42	**2.56**
40-44	2400	4270	**6670**	1.44	2.56	3.99	145144	143572	**288716**	1.65	2.97	**2.31**
45-49	1661	2786	**4447**	0.99	1.67	2.66	115926	112939	**228865**	1.43	2.47	**1.94**
50-54	1152	2078	**3230**	0.69	1.24	1.93	93815	98655	**192470**	1.23	2.11	**1.68**
55-59	1172	1950	**3122**	0.70	1.17	1.87	91223	101690	**192913**	1.28	1.92	**1.62**
60-64	1198	1995	**3193**	0.72	1.19	1.91	93150	105570	**198720**	1.29	1.89	**1.61**
65-69	1399	1891	**3290**	0.84	1.13	1.97	93093	106207	**199300**	1.50	1.78	**1.65**
70-74	1266	1574	**2840**	0.76	0.94	1.70	76573	90083	**166656**	1.65	1.75	**1.70**
75-79	814	969	**1783**	0.49	0.58	1.07	45011	56829	**101840**	1.81	1.71	**1.75**
80-84	724	834	**1558**	0.43	0.50	0.93	36050	51508	**87558**	2.01	1.62	**1.78**
85-89	456	536	**992**	0.27	0.32	0.59	21131	34197	**55328**	2.16	1.57	**1.79**
90-94	177	239	**416**	0.11	0.14	0.25	9147	17225	**26372**	1.94	1.39	**1.58**
95-99	57	60	**117**	0.03	0.04	0.07	2571	5410	**7981**	2.22	1.11	**1.47**
100-104	6	3	**9**	0.00	0.00	0.01	374	665	**1039**	1.60	0.45	**0.87**
105-110	0	0	**0**	0.00	0.00	0.00	87	177	**264**	0.00	0.00	**0.00**
**Total**	**78354**	**88607**	**166961**	**46.93**	**53.07**		**2274349**	**2352834**	**4627183**	**3.45**	**3.77**	**3.61**

eTable 3. Atrial fibrillation prevalence and proportions of patient population by age and sex

	Atrial fibrillation (n)			Percent of atrial fibrillation patients			Total population (n)			Prevalence (%)		
**Age group**	**Male**	**Female**	**Total**	**Male**	**Female**	**Total**	**Male**	**Female**	**Total**	**Male**	**Female**	**Total**
0-4	5	9	**14**	0.01	0.01	0.02	248558	234757	**483315**	0.00	0.00	**0.00**
5-9	16	12	**28**	0.02	0.01	0.03	231281	220106	**451387**	0.01	0.01	**0.01**
10-14	17	10	**27**	0.02	0.01	0.03	199945	190186	**390131**	0.01	0.01	**0.01**
15-19	32	24	**56**	0.04	0.03	0.07	163169	156268	**319437**	0.02	0.02	**0.02**
20-24	84	23	**107**	0.10	0.03	0.12	132509	143680	**276189**	0.06	0.02	**0.04**
25-29	176	72	**248**	0.20	0.08	0.29	156927	159023	**315950**	0.11	0.05	**0.08**
30-34	323	102	**425**	0.38	0.12	0.49	162004	165350	**327354**	0.20	0.06	**0.13**
35-39	478	165	**643**	0.56	0.19	0.75	156661	158737	**315398**	0.31	0.10	**0.20**
40-44	676	254	**930**	0.79	0.30	1.08	145144	143572	**288716**	0.47	0.18	**0.32**
45-49	924	379	**1303**	1.07	0.44	1.51	115926	112939	**228865**	0.80	0.34	**0.57**
50-54	1210	693	**1903**	1.41	0.81	2.21	93815	98655	**192470**	1.29	0.70	**0.99**
55-59	1955	1283	**3238**	2.27	1.49	3.76	91223	101690	**192913**	2.14	1.26	**1.68**
60-64	3344	2311	**5655**	3.88	2.68	6.57	93150	105570	**198720**	3.59	2.19	**2.85**
65-69	5481	4009	**9490**	6.37	4.66	11.02	93093	106207	**199300**	5.89	3.77	**4.76**
70-74	7490	6002	**13492**	8.70	6.97	15.67	76573	90083	**166656**	9.78	6.66	**8.10**
75-79	6206	6272	**12478**	7.21	7.29	14.49	45011	56829	**101840**	13.79	11.04	**12.25**
80-84	6811	8189	**15000**	7.91	9.51	17.42	36050	51508	**87558**	18.89	15.90	**17.13**
85-89	5157	7042	**12199**	5.99	8.18	14.17	21131	34197	**55328**	24.40	20.59	**22.05**
90-94	2598	4084	**6682**	3.02	4.74	7.76	9147	17225	**26372**	28.40	23.71	**25.34**
95-99	669	1257	**1926**	0.78	1.46	2.24	2571	5410	**7981**	26.02	23.23	**24.13**
100-104	84	126	**210**	0.10	0.15	0.24	374	665	**1039**	22.46	18.95	**20.21**
105-110	11	20	**31**	0.01	0.02	0.04	87	177	**264**	12.64	11.30	**11.74**
**Total**	**43747**	**42338**	**86085**	**50.82**	**49.18**		**2274349**	**2352834**	**4627183**	**1.92**	**1.80**	**1.86**

eTable 4. Cerebrovascular accident/transient ischemic attack prevalence and proportions of patient population by age and sex

	s/p Stroke, transient ischemic attack (n)			Percent of stroke, transient ischemic attack patients			Total population (n)			Prevalence (%)		
**Age group**	**Male**	**Female**	**Total**	**Male**	**Female**	**Total**	**Male**	**Female**	**Total**	**Male**	**Female**	**Total**
0-4	176	89	**265**	0.15	0.07	0.22	248558	234757	**483315**	0.07	0.04	**0.05**
5-9	226	181	**407**	0.19	0.15	0.34	231281	220106	**451387**	0.10	0.08	**0.09**
10-14	284	195	**479**	0.24	0.16	0.40	199945	190186	**390131**	0.14	0.10	**0.12**
15-19	352	245	**597**	0.29	0.20	0.50	163169	156268	**319437**	0.22	0.16	**0.19**
20-24	402	227	**629**	0.33	0.19	0.52	132509	143680	**276189**	0.30	0.16	**0.23**
25-29	356	290	**646**	0.30	0.24	0.54	156927	159023	**315950**	0.23	0.18	**0.20**
30-34	441	457	**898**	0.37	0.38	0.75	162004	165350	**327354**	0.27	0.28	**0.27**
35-39	644	629	**1273**	0.53	0.52	1.06	156661	158737	**315398**	0.41	0.40	**0.40**
40-44	987	965	**1952**	0.82	0.80	1.62	145144	143572	**288716**	0.68	0.67	**0.68**
45-49	1524	1183	**2707**	1.26	0.98	2.25	115926	112939	**228865**	1.31	1.05	**1.18**
50-54	2384	1914	**4298**	1.98	1.59	3.57	93815	98655	**192470**	2.54	1.94	**2.23**
55-59	4124	3148	**7272**	3.42	2.61	6.04	91223	101690	**192913**	4.52	3.10	**3.77**
60-64	6394	4867	**11261**	5.31	4.04	9.35	93150	105570	**198720**	6.86	4.61	**5.67**
65-69	9163	6725	**15888**	7.61	5.58	13.19	93093	106207	**199300**	9.84	6.33	**7.97**
70-74	9900	8074	**17974**	8.22	6.70	14.92	76573	90083	**166656**	12.93	8.96	**10.79**
75-79	7886	7306	**15192**	6.55	6.06	12.61	45011	56829	**101840**	17.52	12.86	**14.92**
80-84	7652	8986	**16638**	6.35	7.46	13.81	36050	51508	**87558**	21.23	17.45	**19.00**
85-89	5327	7553	**12880**	4.42	6.27	10.69	21131	34197	**55328**	25.21	22.09	**23.28**
90-94	2577	4268	**6845**	2.14	3.54	5.68	9147	17225	**26372**	28.17	24.78	**25.96**
95-99	719	1383	**2102**	0.60	1.15	1.74	2571	5410	**7981**	27.97	25.56	**26.34**
100-104	81	152	**233**	0.07	0.13	0.19	374	665	**1039**	21.66	22.86	**22.43**
105-110	13	26	**39**	0.01	0.02	0.03	87	177	**264**	14.94	14.69	**14.77**
**Total**	**61612**	**58863**	**120475**	**51.14**	**48.86**		**2274349**	**2352834**	**4627183**	**2.71**	**2.50**	**2.60**

eTable 5. Chronic obstructive pulmonary disease (including bronchiectasis) prevalence and proportions of patient population by age and sex

	Chronic obstructive pulmonary disease (COPD)			Percent of COPD patients			Total population (n)			Prevalence (%)		
**Age group**	**Male**	**Female**	**Total**	**Male**	**Female**	**Total**	**Male**	**Female**	**Total**	**Male**	**Female**	**Total**
0-4	239	129	**368**	0.28	0.15	0.44	248558	234757	**483315**	0.10	0.05	**0.08**
5-9	190	143	**333**	0.23	0.17	0.40	231281	220106	**451387**	0.08	0.06	**0.07**
10-14	290	145	**435**	0.34	0.17	0.52	199945	190186	**390131**	0.15	0.08	**0.11**
15-19	440	272	**712**	0.52	0.32	0.85	163169	156268	**319437**	0.27	0.17	**0.22**
20-24	338	249	**587**	0.40	0.30	0.70	132509	143680	**276189**	0.26	0.17	**0.21**
25-29	339	234	**573**	0.40	0.28	0.68	156927	159023	**315950**	0.22	0.15	**0.18**
30-34	415	295	**710**	0.49	0.35	0.84	162004	165350	**327354**	0.26	0.18	**0.22**
35-39	647	465	**1112**	0.77	0.55	1.32	156661	158737	**315398**	0.41	0.29	**0.35**
40-44	978	675	**1653**	1.16	0.80	1.97	145144	143572	**288716**	0.67	0.47	**0.57**
45-49	1497	918	**2415**	1.78	1.09	2.87	115926	112939	**228865**	1.29	0.81	**1.06**
50-54	2443	1485	**3928**	2.91	1.77	4.67	93815	98655	**192470**	2.60	1.51	**2.04**
55-59	4120	2698	**6818**	4.90	3.21	8.11	91223	101690	**192913**	4.52	2.65	**3.53**
60-64	5799	4213	**10012**	6.90	5.01	11.91	93150	105570	**198720**	6.23	3.99	**5.04**
65-69	7138	5351	**12489**	8.49	6.36	14.85	93093	106207	**199300**	7.67	5.04	**6.27**
70-74	6998	5501	**12499**	8.32	6.54	14.86	76573	90083	**166656**	9.14	6.11	**7.50**
75-79	5417	4238	**9655**	6.44	5.04	11.48	45011	56829	**101840**	12.03	7.46	**9.48**
80-84	4755	4609	**9364**	5.65	5.48	11.14	36050	51508	**87558**	13.19	8.95	**10.69**
85-89	3091	3329	**6420**	3.68	3.96	7.63	21131	34197	**55328**	14.63	9.73	**11.60**
90-94	1261	1707	**2968**	1.50	2.03	3.53	9147	17225	**26372**	13.79	9.91	**11.25**
95-99	333	553	**886**	0.40	0.66	1.05	2571	5410	**7981**	12.95	10.22	**11.10**
100-104	54	67	**121**	0.06	0.08	0.14	374	665	**1039**	14.44	10.08	**11.65**
105-110	12	17	**29**	0.01	0.02	0.03	87	177	**264**	13.79	9.60	**10.98**
**Total**	**46794**	**37293**	**84087**	**55.65**	**44.35**		**2274349**	**2352834**	**4627183**	**2.06**	**1.59**	**1.82**

eTable 6. Congestive heart failure (CHF) prevalence and proportions of patient population by age and sex

	Congestive heart failure (CHF) (n)			Percent of CHF patients			Total population (n)			Prevalence (%)		
**Age group**	**Male**	**Female**	**Total**	**Male**	**Female**	**Total**	**Male**	**Female**	**Total**	**Male**	**Female**	**Total**
0-4	62	62	**124**	0.11	0.11	0.23	248558	234757	**483315**	0.02	0.03	**0.03**
5-9	56	67	**123**	0.10	0.12	0.22	231281	220106	**451387**	0.02	0.03	**0.03**
10-14	54	44	**98**	0.10	0.08	0.18	199945	190186	**390131**	0.03	0.02	**0.03**
15-19	53	42	**95**	0.10	0.08	0.17	163169	156268	**319437**	0.03	0.03	**0.03**
20-24	71	46	**117**	0.13	0.08	0.21	132509	143680	**276189**	0.05	0.03	**0.04**
25-29	73	46	**119**	0.13	0.08	0.22	156927	159023	**315950**	0.05	0.03	**0.04**
30-34	98	78	**176**	0.18	0.14	0.32	162004	165350	**327354**	0.06	0.05	**0.05**
35-39	178	131	**309**	0.32	0.24	0.56	156661	158737	**315398**	0.11	0.08	**0.10**
40-44	362	181	**543**	0.66	0.33	0.99	145144	143572	**288716**	0.25	0.13	**0.19**
45-49	638	260	**898**	1.16	0.47	1.64	115926	112939	**228865**	0.55	0.23	**0.39**
50-54	1112	458	**1570**	2.03	0.83	2.86	93815	98655	**192470**	1.19	0.46	**0.82**
55-59	1965	778	**2743**	3.58	1.42	5.00	91223	101690	**192913**	2.15	0.77	**1.42**
60-64	3124	1331	**4455**	5.69	2.43	8.12	93150	105570	**198720**	3.35	1.26	**2.24**
65-69	4308	2089	**6397**	7.85	3.81	11.66	93093	106207	**199300**	4.63	1.97	**3.21**
70-74	4887	2909	**7796**	8.91	5.30	14.21	76573	90083	**166656**	6.38	3.23	**4.68**
75-79	3998	3160	**7158**	7.29	5.76	13.05	45011	56829	**101840**	8.88	5.56	**7.03**
80-84	4219	4483	**8702**	7.69	8.17	15.86	36050	51508	**87558**	11.70	8.70	**9.94**
85-89	3251	4274	**7525**	5.92	7.79	13.71	21131	34197	**55328**	15.38	12.50	**13.60**
90-94	1639	2687	**4326**	2.99	4.90	7.88	9147	17225	**26372**	17.92	15.60	**16.40**
95-99	504	897	**1401**	0.92	1.63	2.55	2571	5410	**7981**	19.60	16.58	**17.55**
100-104	49	113	**162**	0.09	0.21	0.30	374	665	**1039**	13.10	16.99	**15.59**
105-110	10	24	**34**	0.02	0.04	0.06	87	177	**264**	11.49	13.56	**12.88**
**Total**	**30711**	**24160**	**54871**	**55.97**	**44.03**		**2274349**	**2352834**	**4627183**	**1.35**	**1.03**	**1.19**

eTable 7. Dementia (age 60 years and above) prevalence and proportions of patient population by age and sex

	Dementia (n)			Percent of dementia patients			Total population (n)			Prevalence (%)		
Age group	Male	Female	Total	Male	Female	Total	Male	Female	Total	Male	Female	Total
60-64	572	471	**1043**	1.18	0.97	2.14	93150	105570	**198720**	0.61	0.45	**0.52**
65-69	1169	1078	**2247**	2.40	2.22	4.62	93093	106207	**199300**	1.26	1.01	**1.13**
70-74	1935	2160	**4095**	3.98	4.44	8.41	76573	90083	**166656**	2.53	2.40	**2.46**
75-79	2694	3987	**6681**	5.54	8.19	13.73	45011	56829	**101840**	5.99	7.02	**6.56**
80-84	3868	7681	**11549**	7.95	15.78	23.73	36050	51508	**87558**	10.73	14.91	**13.19**
85-89	3774	8622	**12396**	7.76	17.72	25.47	21131	34197	**55328**	17.86	25.21	**22.40**
90-94	2071	5632	**7703**	4.26	11.57	15.83	9147	17225	**26372**	22.64	32.70	**29.21**
95-99	683	1924	**2607**	1.40	3.95	5.36	2571	5410	**7981**	26.57	35.56	**32.67**
100-104	74	225	**299**	0.15	0.46	0.61	374	665	**1039**	19.79	33.83	**28.78**
105-110	17	28	**45**	0.03	0.06	0.09	87	177	**264**	19.54	15.82	**17.05**
**Total**	**16857**	**31808**	**48665**	**34.64**	**65.36**		**377187**	**467871**	**845058**	**4.47**	**6.80**	**5.76**

eTable 8. Depression and bipolar disease prevalence and proportions of patient population by age and sex

	Depression (n)			Percent of depression patients			Total population (n)			Prevalence (%)		
Age group	Male	Female	Total	Male	Female	Total	Male	Female	Total	Male	Female	Total
0-4	19	10	**29**	0.01	0.01	0.01	248558	234757	**483315**	0.01	0.00	**0.01**
5-9	58	34	**92**	0.03	0.02	0.05	231281	220106	**451387**	0.03	0.02	**0.02**
10-14	356	265	**621**	0.18	0.14	0.32	199945	190186	**390131**	0.18	0.14	**0.16**
15-19	832	1011	**1843**	0.43	0.52	0.95	163169	156268	**319437**	0.51	0.65	**0.58**
20-24	1457	1815	**3272**	0.75	0.93	1.69	132509	143680	**276189**	1.10	1.26	**1.18**
25-29	2449	3369	**5818**	1.26	1.74	3.00	156927	159023	**315950**	1.56	2.12	**1.84**
30-34	3516	5164	**8680**	1.81	2.66	4.47	162004	165350	**327354**	2.17	3.12	**2.65**
35-39	4437	6824	**11261**	2.29	3.51	5.80	156661	158737	**315398**	2.83	4.30	**3.57**
40-44	5073	7600	**12673**	2.61	3.91	6.53	145144	143572	**288716**	3.50	5.29	**4.39**
45-49	4777	7559	**12336**	2.46	3.89	6.35	115926	112939	**228865**	4.12	6.69	**5.39**
50-54	4965	8153	**13118**	2.56	4.20	6.76	93815	98655	**192470**	5.29	8.26	**6.82**
55-59	5861	9839	**15700**	3.02	5.07	8.09	91223	101690	**192913**	6.42	9.68	**8.14**
60-64	6760	11589	**18349**	3.48	5.97	9.45	93150	105570	**198720**	7.26	10.98	**9.23**
65-69	7154	12138	**19292**	3.68	6.25	9.94	93093	106207	**199300**	7.68	11.43	**9.68**
70-74	5901	11413	**17314**	3.04	5.88	8.92	76573	90083	**166656**	7.71	12.67	**10.39**
75-79	4532	9674	**14206**	2.33	4.98	7.32	45011	56829	**101840**	10.07	17.02	**13.95**
80-84	4603	11968	**16571**	2.37	6.16	8.53	36050	51508	**87558**	12.77	23.24	**18.93**
85-89	3556	9880	**13436**	1.83	5.09	6.92	21131	34197	**55328**	16.83	28.89	**24.28**
90-94	1773	5473	**7246**	0.91	2.82	3.73	9147	17225	**26372**	19.38	31.77	**27.48**
95-99	519	1543	**2062**	0.27	0.79	1.06	2571	5410	**7981**	20.19	28.52	**25.84**
100-104	41	172	**213**	0.02	0.09	0.11	374	665	**1039**	10.96	25.86	**20.50**
105-110	6	19	**25**	0.00	0.01	0.01	87	177	**264**	6.90	10.73	**9.47**
**Total**	**68645**	**125512**	**194157**	**35.36**	**64.64**		**2274349**	**2352834**	**4627183**	**3.02**	**5.33**	**4.20**

eTable 9. Diabetes mellitus prevalence and proportions of patient population by age and sex

	Diabetes mellitus (n)			Percent of diabetes mellitus patients			Total population (n)			Prevalence (%)		
**Age group**	**Male**	**Female**	**Total**	**Male**	**Female**	**Total**	**Male**	**Female**	**Total**	**Male**	**Female**	**Total**
0-4	52	41	**93**	0.01	0.01	0.02	248558	234757	**483315**	0.02	0.02	**0.02**
5-9	189	186	**375**	0.05	0.05	0.09	231281	220106	**451387**	0.08	0.08	**0.08**
10-14	468	437	**905**	0.12	0.11	0.23	199945	190186	**390131**	0.23	0.23	**0.23**
15-19	742	692	**1434**	0.19	0.17	0.36	163169	156268	**319437**	0.45	0.44	**0.45**
20-24	782	802	**1584**	0.20	0.20	0.40	132509	143680	**276189**	0.59	0.56	**0.57**
25-29	1071	1180	**2251**	0.27	0.29	0.56	156927	159023	**315950**	0.68	0.74	**0.71**
30-34	2009	2004	**4013**	0.50	0.50	1.00	162004	165350	**327354**	1.24	1.21	**1.23**
35-39	3591	3172	**6763**	0.90	0.79	1.69	156661	158737	**315398**	2.29	2.00	**2.14**
40-44	6930	5030	**11960**	1.73	1.26	2.99	145144	143572	**288716**	4.77	3.50	**4.14**
45-49	10595	7641	**18236**	2.65	1.91	4.56	115926	112939	**228865**	9.14	6.77	**7.97**
50-54	14678	11903	**26581**	3.67	2.97	6.64	93815	98655	**192470**	15.65	12.07	**13.81**
55-59	20678	18094	**38772**	5.17	4.52	9.69	91223	101690	**192913**	22.67	17.79	**20.10**
60-64	26961	24942	**51903**	6.73	6.23	12.97	93150	105570	**198720**	28.94	23.63	**26.12**
65-69	32947	31194	**64141**	8.23	7.79	16.02	93093	106207	**199300**	35.39	29.37	**32.18**
70-74	30599	30637	**61236**	7.64	7.65	15.30	76573	90083	**166656**	39.96	34.01	**36.74**
75-79	19519	21782	**41301**	4.88	5.44	10.32	45011	56829	**101840**	43.36	38.33	**40.55**
80-84	15409	20499	**35908**	3.85	5.12	8.97	36050	51508	**87558**	42.74	39.80	**41.01**
85-89	8555	12960	**21515**	2.14	3.24	5.37	21131	34197	**55328**	40.49	37.90	**38.89**
90-94	3288	5512	**8800**	0.82	1.38	2.20	9147	17225	**26372**	35.95	32.00	**33.37**
95-99	791	1483	**2274**	0.20	0.37	0.57	2571	5410	**7981**	30.77	27.41	**28.49**
100-104	103	139	**242**	0.03	0.03	0.06	374	665	**1039**	27.54	20.90	**23.29**
105-110	8	24	**32**	0.00	0.01	0.01	87	177	**264**	9.20	13.56	**12.12**
**Total**	**199965**	**200354**	**400319**	**49.95**	**50.05**		**2274349**	**2352834**	**4627183**	**8.79**	**8.52**	**8.65**

eTable 10. Chronic dialysis prevalence and proportions of patient population by age and sex

	Chronic dialysis (n)			Percent of chronic dialysis patients			Total population (n)			Prevalence (%)		
**Age group**	**Male**	**Female**	**Total**	**Male**	**Female**	**Total**	**Male**	**Female**	**Total**	**Male**	**Female**	**Total**
0-4	8	6	**14**	0.13	0.09	0.22	248558	234757	**483315**	0.00	0.00	**0.00**
5-9	16	9	**25**	0.25	0.14	0.39	231281	220106	**451387**	0.01	0.00	**0.01**
10-14	17	17	**34**	0.27	0.27	0.53	199945	190186	**390131**	0.01	0.01	**0.01**
15-19	27	17	**44**	0.42	0.27	0.69	163169	156268	**319437**	0.02	0.01	**0.01**
20-24	52	34	**86**	0.82	0.53	1.35	132509	143680	**276189**	0.04	0.02	**0.03**
25-29	63	36	**99**	0.99	0.57	1.56	156927	159023	**315950**	0.04	0.02	**0.03**
30-34	88	59	**147**	1.38	0.93	2.31	162004	165350	**327354**	0.05	0.04	**0.04**
35-39	126	77	**203**	1.98	1.21	3.19	156661	158737	**315398**	0.08	0.05	**0.06**
40-44	173	81	**254**	2.72	1.27	3.99	145144	143572	**288716**	0.12	0.06	**0.09**
45-49	210	115	**325**	3.30	1.81	5.11	115926	112939	**228865**	0.18	0.10	**0.14**
50-54	244	169	**413**	3.83	2.66	6.49	93815	98655	**192470**	0.26	0.17	**0.21**
55-59	332	183	**515**	5.22	2.88	8.09	91223	101690	**192913**	0.36	0.18	**0.27**
60-64	483	243	**726**	7.59	3.82	11.41	93150	105570	**198720**	0.52	0.23	**0.37**
65-69	570	370	**940**	8.96	5.81	14.77	93093	106207	**199300**	0.61	0.35	**0.47**
70-74	572	353	**925**	8.99	5.55	14.53	76573	90083	**166656**	0.75	0.39	**0.56**
75-79	363	277	**640**	5.70	4.35	10.06	45011	56829	**101840**	0.81	0.49	**0.63**
80-84	339	246	**585**	5.33	3.87	9.19	36050	51508	**87558**	0.94	0.48	**0.67**
85-89	175	132	**307**	2.75	2.07	4.82	21131	34197	**55328**	0.83	0.39	**0.55**
90-94	42	28	**70**	0.66	0.44	1.10	9147	17225	**26372**	0.46	0.16	**0.27**
95-99	9	3	**12**	0.14	0.05	0.19	2571	5410	**7981**	0.35	0.06	**0.15**
100-104	0	0	**0**	0.00	0.00	0.00	374	665	**1039**	0.00	0.00	**0.00**
105-110	0	0	**0**	0.00	0.00	0.00	87	177	**264**	0.00	0.00	**0.00**
**Total**	**3909**	**2455**	**6364**	**61.42**	**38.58**		**2274349**	**2352834**	**4627183**	**0.17**	**0.10**	**0.14**

eTable 11. Epilepsy prevalence and proportions of patient population by age and sex

	Epilepsy (n)			Percent of epilepsy patients			Total population (n)			Prevalence (%)		
**Age group**	**Male**	**Female**	**Total**	**Male**	**Female**	**Total**	**Male**	**Female**	**Total**	**Male**	**Female**	**Total**
0-4	465	424	**889**	0.94	0.86	1.80	248558	234757	**483315**	0.19	0.18	**0.18**
5-9	1220	922	**2142**	2.47	1.87	4.33	231281	220106	**451387**	0.53	0.42	**0.47**
10-14	1642	1272	**2914**	3.32	2.57	5.90	199945	190186	**390131**	0.82	0.67	**0.75**
15-19	1720	1407	**3127**	3.48	2.85	6.33	163169	156268	**319437**	1.05	0.90	**0.98**
20-24	1773	1585	**3358**	3.59	3.21	6.79	132509	143680	**276189**	1.34	1.10	**1.22**
25-29	2008	1754	**3762**	4.06	3.55	7.61	156927	159023	**315950**	1.28	1.10	**1.19**
30-34	2083	1844	**3927**	4.21	3.73	7.95	162004	165350	**327354**	1.29	1.12	**1.20**
35-39	1993	1750	**3743**	4.03	3.54	7.57	156661	158737	**315398**	1.27	1.10	**1.19**
40-44	1797	1605	**3402**	3.64	3.25	6.88	145144	143572	**288716**	1.24	1.12	**1.18**
45-49	1655	1349	**3004**	3.35	2.73	6.08	115926	112939	**228865**	1.43	1.19	**1.31**
50-54	1457	1302	**2759**	2.95	2.63	5.58	93815	98655	**192470**	1.55	1.32	**1.43**
55-59	1425	1324	**2749**	2.88	2.68	5.56	91223	101690	**192913**	1.56	1.30	**1.42**
60-64	1481	1408	**2889**	3.00	2.85	5.85	93150	105570	**198720**	1.59	1.33	**1.45**
65-69	1528	1572	**3100**	3.09	3.18	6.27	93093	106207	**199300**	1.64	1.48	**1.56**
70-74	1349	1360	**2709**	2.73	2.75	5.48	76573	90083	**166656**	1.76	1.51	**1.63**
75-79	825	884	**1709**	1.67	1.79	3.46	45011	56829	**101840**	1.83	1.56	**1.68**
80-84	676	901	**1577**	1.37	1.82	3.19	36050	51508	**87558**	1.88	1.75	**1.80**
85-89	390	616	**1006**	0.79	1.25	2.04	21131	34197	**55328**	1.85	1.80	**1.82**
90-94	186	315	**501**	0.38	0.64	1.01	9147	17225	**26372**	2.03	1.83	**1.90**
95-99	48	90	**138**	0.10	0.18	0.28	2571	5410	**7981**	1.87	1.66	**1.73**
100-104	5	10	**15**	0.01	0.02	0.03	374	665	**1039**	1.34	1.50	**1.44**
105-110	0	2	**2**	0.00	0.00	0.00	87	177	**264**	0.00	1.13	**0.76**
**Total**	**25726**	**23696**	**49422**	**52.05**	**47.95**		**2274349**	**2352834**	**4627183**	**1.13**	**1.01**	**1.07**

eTable 12. Gastroesophageal reflux, gastritis, duodenitis prevalence and proportions of patient population by age and sex

	Reflux esophagitis, gastritis, duodenitis (n)			Percent of Reflux Esophagitis, Gastritis, Deudenitis patients			Total population (n)			Prevalence (%)		
**Age group**	**Male**	**Female**	**Total**	**Male**	**Female**	**Total**	**Male**	**Female**	**Total**	**Male**	**Female**	**Total**
0-4	2398	2203	**4601**	0.94	0.87	1.81	248558	234757	**483315**	0.96	0.94	**0.95**
5-9	2770	2382	**5152**	1.09	0.94	2.03	231281	220106	**451387**	1.20	1.08	**1.14**
10-14	2448	2277	**4725**	0.96	0.90	1.86	199945	190186	**390131**	1.22	1.20	**1.21**
15-19	2088	2285	**4373**	0.82	0.90	1.72	163169	156268	**319437**	1.28	1.46	**1.37**
20-24	1702	2718	**4420**	0.67	1.07	1.74	132509	143680	**276189**	1.28	1.89	**1.60**
25-29	2580	4033	**6613**	1.02	1.59	2.60	156927	159023	**315950**	1.64	2.54	**2.09**
30-34	4078	5373	**9451**	1.60	2.11	3.72	162004	165350	**327354**	2.52	3.25	**2.89**
35-39	5372	6139	**11511**	2.11	2.42	4.53	156661	158737	**315398**	3.43	3.87	**3.65**
40-44	6811	7081	**13892**	2.68	2.79	5.47	145144	143572	**288716**	4.69	4.93	**4.81**
45-49	7102	7281	**14383**	2.80	2.87	5.66	115926	112939	**228865**	6.13	6.45	**6.28**
50-54	7530	8437	**15967**	2.96	3.32	6.28	93815	98655	**192470**	8.03	8.55	**8.30**
55-59	8993	10773	**19766**	3.54	4.24	7.78	91223	101690	**192913**	9.86	10.59	**10.25**
60-64	10792	13305	**24097**	4.25	5.24	9.48	93150	105570	**198720**	11.59	12.60	**12.13**
65-69	12459	15711	**28170**	4.90	6.18	11.09	93093	106207	**199300**	13.38	14.79	**14.13**
70-74	12002	15956	**27958**	4.72	6.28	11.00	76573	90083	**166656**	15.67	17.71	**16.78**
75-79	7846	11376	**19222**	3.09	4.48	7.56	45011	56829	**101840**	17.43	20.02	**18.87**
80-84	7239	11827	**19066**	2.85	4.65	7.50	36050	51508	**87558**	20.08	22.96	**21.78**
85-89	4444	8356	**12800**	1.75	3.29	5.04	21131	34197	**55328**	21.03	24.43	**23.13**
90-94	2003	4031	**6034**	0.79	1.59	2.37	9147	17225	**26372**	21.90	23.40	**22.88**
95-99	517	1159	**1676**	0.20	0.46	0.66	2571	5410	**7981**	20.11	21.42	**21.00**
100-104	68	122	**190**	0.03	0.05	0.07	374	665	**1039**	18.18	18.35	**18.29**
105-110	8	19	**27**	0.00	0.01	0.01	87	177	**264**	9.20	10.73	**10.23**
**Total**	**111250**	**142844**	**254094**	**43.78**	**56.22**		**2274349**	**2352834**	**4627183**	**4.89**	**6.07**	**5.49**

eTable 13. Glaucoma prevalence and proportions of patient population by age and sex

	Glaucoma (n)			Percent of Glaucoma Patients			Total population (n)			Prevalence (%)		
Age group	Male	Female	Total	Male	Female	Total	Male	Female	Total	Male	Female	Total
0-4	47	54	**101**	0.06	0.06	0.12	248558	234757	**483315**	0.02	0.02	**0.02**
5-9	86	73	**159**	0.10	0.09	0.19	231281	220106	**451387**	0.04	0.03	**0.04**
10-14	138	119	**257**	0.16	0.14	0.31	199945	190186	**390131**	0.07	0.06	**0.07**
15-19	189	187	**376**	0.22	0.22	0.45	163169	156268	**319437**	0.12	0.12	**0.12**
20-24	208	265	**473**	0.25	0.31	0.56	132509	143680	**276189**	0.16	0.18	**0.17**
25-29	266	350	**616**	0.32	0.42	0.73	156927	159023	**315950**	0.17	0.22	**0.19**
30-34	340	422	**762**	0.40	0.50	0.90	162004	165350	**327354**	0.21	0.26	**0.23**
35-39	412	478	**890**	0.49	0.57	1.06	156661	158737	**315398**	0.26	0.30	**0.28**
40-44	576	540	**1116**	0.68	0.64	1.33	145144	143572	**288716**	0.40	0.38	**0.39**
45-49	689	637	**1326**	0.82	0.76	1.57	115926	112939	**228865**	0.59	0.56	**0.58**
50-54	973	976	**1949**	1.16	1.16	2.31	93815	98655	**192470**	1.04	0.99	**1.01**
55-59	1539	1479	**3018**	1.83	1.76	3.58	91223	101690	**192913**	1.69	1.45	**1.56**
60-64	2574	2784	**5358**	3.06	3.31	6.36	93150	105570	**198720**	2.76	2.64	**2.70**
65-69	4424	4670	**9094**	5.25	5.55	10.80	93093	106207	**199300**	4.75	4.40	**4.56**
70-74	6206	6779	**12985**	7.37	8.05	15.42	76573	90083	**166656**	8.10	7.53	**7.79**
75-79	5420	6254	**11674**	6.44	7.43	13.86	45011	56829	**101840**	12.04	11.00	**11.46**
80-84	6196	8136	**14332**	7.36	9.66	17.02	36050	51508	**87558**	17.19	15.80	**16.37**
85-89	4524	6629	**11153**	5.37	7.87	13.25	21131	34197	**55328**	21.41	19.38	**20.16**
90-94	2337	3854	**6191**	2.78	4.58	7.35	9147	17225	**26372**	25.55	22.37	**23.48**
95-99	675	1391	**2066**	0.80	1.65	2.45	2571	5410	**7981**	26.25	25.71	**25.89**
100-104	107	163	**270**	0.13	0.19	0.32	374	665	**1039**	28.61	24.51	**25.99**
105-110	14	21	**35**	0.02	0.02	0.04	87	177	**264**	16.09	11.86	**13.26**
**Total**	**37940**	**46261**	**84201**	**45.06**	**54.94**		**2274349**	**2352834**	**4627183**	**1.67**	**1.97**	**1.82**

eTable 14. Gout prevalence and proportions of patient population by age and sex

	Gout (n)			Percent of gout patients			Total population (n)			Prevalence (%)		
**Age group**	**Male**	**Female**	**Total**	**Male**	**Female**	**Total**	**Male**	**Female**	**Total**	**Male**	**Female**	**Total**
0-4	2	2	**4**	0.01	0.01	0.01	248558	234757	**483315**	0.00	0.00	**0.00**
5-9	3	1	**4**	0.01	0.00	0.01	231281	220106	**451387**	0.00	0.00	**0.00**
10-14	4	2	**6**	0.01	0.01	0.02	199945	190186	**390131**	0.00	0.00	**0.00**
15-19	14	7	**21**	0.05	0.03	0.08	163169	156268	**319437**	0.01	0.00	**0.01**
20-24	58	20	**78**	0.21	0.07	0.28	132509	143680	**276189**	0.04	0.01	**0.03**
25-29	198	30	**228**	0.71	0.11	0.82	156927	159023	**315950**	0.13	0.02	**0.07**
30-34	463	36	**499**	1.66	0.13	1.79	162004	165350	**327354**	0.29	0.02	**0.15**
35-39	794	59	**853**	2.85	0.21	3.06	156661	158737	**315398**	0.51	0.04	**0.27**
40-44	1088	72	**1160**	3.90	0.26	4.16	145144	143572	**288716**	0.75	0.05	**0.40**
45-49	1339	107	**1446**	4.80	0.38	5.18	115926	112939	**228865**	1.16	0.09	**0.63**
50-54	1409	164	**1573**	5.05	0.59	5.64	93815	98655	**192470**	1.50	0.17	**0.82**
55-59	1973	248	**2221**	7.07	0.89	7.96	91223	101690	**192913**	2.16	0.24	**1.15**
60-64	2718	389	**3107**	9.75	1.39	11.14	93150	105570	**198720**	2.92	0.37	**1.56**
65-69	3241	565	**3806**	11.62	2.03	13.65	93093	106207	**199300**	3.48	0.53	**1.91**
70-74	3341	699	**4040**	11.98	2.51	14.49	76573	90083	**166656**	4.36	0.78	**2.42**
75-79	2222	575	**2797**	7.97	2.06	10.03	45011	56829	**101840**	4.94	1.01	**2.75**
80-84	2202	783	**2985**	7.90	2.81	10.70	36050	51508	**87558**	6.11	1.52	**3.41**
85-89	1345	563	**1908**	4.82	2.02	6.84	21131	34197	**55328**	6.37	1.65	**3.45**
90-94	602	287	**889**	2.16	1.03	3.19	9147	17225	**26372**	6.58	1.67	**3.37**
95-99	145	89	**234**	0.52	0.32	0.84	2571	5410	**7981**	5.64	1.65	**2.93**
100-104	18	6	**24**	0.06	0.02	0.09	374	665	**1039**	4.81	0.90	**2.31**
105-110	3	4	**7**	0.01	0.01	0.03	87	177	**264**	3.45	2.26	**2.65**
**Total**	**23182**	**4708**	**27890**	**83.12**	**16.88**		**2274349**	**2352834**	**4627183**	**1.02**	**0.20**	**0.60**

eTable 15. Hypertension prevalence and proportions of patient population by age and sex

	Hypertension (n)			Percent of hypertension patients			Total population (n)			Prevalence (%)		
**Age group**	**Male**	**Female**	**Total**	**Male**	**Female**	**Total**	**Male**	**Female**	**Total**	**Male**	**Female**	**Total**
0-4	52	45	**97**	0.01	0.01	0.02	248558	234757	**483315**	0.02	0.02	**0.02**
5-9	83	75	**158**	0.01	0.01	0.03	231281	220106	**451387**	0.04	0.03	**0.04**
10-14	149	110	**259**	0.02	0.02	0.04	199945	190186	**390131**	0.07	0.06	**0.07**
15-19	334	188	**522**	0.06	0.03	0.09	163169	156268	**319437**	0.20	0.12	**0.16**
20-24	691	353	**1044**	0.12	0.06	0.17	132509	143680	**276189**	0.52	0.25	**0.38**
25-29	1185	626	**1811**	0.20	0.10	0.30	156927	159023	**315950**	0.76	0.39	**0.57**
30-34	2137	1278	**3415**	0.36	0.21	0.57	162004	165350	**327354**	1.32	0.77	**1.04**
35-39	3742	2456	**6198**	0.63	0.41	1.04	156661	158737	**315398**	2.39	1.55	**1.97**
40-44	7230	5135	**12365**	1.21	0.86	2.07	145144	143572	**288716**	4.98	3.58	**4.28**
45-49	11685	9034	**20719**	1.95	1.51	3.47	115926	112939	**228865**	10.08	8.00	**9.05**
50-54	16467	14573	**31040**	2.75	2.44	5.19	93815	98655	**192470**	17.55	14.77	**16.13**
55-59	25246	23605	**48851**	4.22	3.95	8.17	91223	101690	**192913**	27.68	23.21	**25.32**
60-64	35655	34605	**70260**	5.96	5.79	11.75	93150	105570	**198720**	38.28	32.78	**35.36**
65-69	46706	46410	**93116**	7.81	7.76	15.58	93093	106207	**199300**	50.17	43.70	**46.72**
70-74	46848	51364	**98212**	7.84	8.59	16.43	76573	90083	**166656**	61.18	57.02	**58.93**
75-79	30662	37939	**68601**	5.13	6.35	11.48	45011	56829	**101840**	68.12	66.76	**67.36**
80-84	27047	39406	**66453**	4.52	6.59	11.12	36050	51508	**87558**	75.03	76.50	**75.90**
85-89	16861	28052	**44913**	2.82	4.69	7.51	21131	34197	**55328**	79.79	82.03	**81.18**
90-94	7435	14727	**22162**	1.24	2.46	3.71	9147	17225	**26372**	81.28	85.50	**84.04**
95-99	2079	4599	**6678**	0.35	0.77	1.12	2571	5410	**7981**	80.86	85.01	**83.67**
100-104	252	516	**768**	0.04	0.09	0.13	374	665	**1039**	67.38	77.59	**73.92**
105-110	35	85	**120**	0.01	0.01	0.02	87	177	**264**	40.23	48.02	**45.45**
**Total**	**282581**	**315181**	**597762**	**47.27**	**52.73**		**2274349**	**2352834**	**4627183**	**12.42**	**13.40**	**12.92**

eTable 16. Ischemic heart disease prevalence and proportions of patient population by age and sex

	Ischemic heart disease (n)			Percent of ischemic heart disease patients			Total population			Prevalence (%)		
Age group	Male	Female	Total	Male	Female	Total	Male	Female	Total	Male	Female	Total
0-4	17	12	**29**	0.01	0.01	0.01	248558	234757	**483315**	0.01	0.01	**0.01**
5-9	31	24	**55**	0.01	0.01	0.03	231281	220106	**451387**	0.01	0.01	**0.01**
10-14	24	11	**35**	0.01	0.01	0.02	199945	190186	**390131**	0.01	0.01	**0.01**
15-19	87	74	**161**	0.04	0.04	0.08	163169	156268	**319437**	0.05	0.05	**0.05**
20-24	61	65	**126**	0.03	0.03	0.06	132509	143680	**276189**	0.05	0.05	**0.05**
25-29	128	82	**210**	0.06	0.04	0.10	156927	159023	**315950**	0.08	0.05	**0.07**
30-34	342	198	**540**	0.16	0.09	0.26	162004	165350	**327354**	0.21	0.12	**0.16**
35-39	819	296	**1115**	0.39	0.14	0.53	156661	158737	**315398**	0.52	0.19	**0.35**
40-44	1987	508	**2495**	0.95	0.24	1.19	145144	143572	**288716**	1.37	0.35	**0.86**
45-49	3936	887	**4823**	1.88	0.42	2.31	115926	112939	**228865**	3.40	0.79	**2.11**
50-54	7016	1540	**8556**	3.36	0.74	4.09	93815	98655	**192470**	7.48	1.56	**4.45**
55-59	11579	2994	**14573**	5.54	1.43	6.97	91223	101690	**192913**	12.69	2.94	**7.55**
60-64	17611	5523	**23134**	8.43	2.64	11.07	93150	105570	**198720**	18.91	5.23	**11.64**
65-69	23369	8306	**31675**	11.18	3.97	15.15	93093	106207	**199300**	25.10	7.82	**15.89**
70-74	24083	10635	**34718**	11.52	5.09	16.61	76573	90083	**166656**	31.45	11.81	**20.83**
75-79	16629	9518	**26147**	7.96	4.55	12.51	45011	56829	**101840**	36.94	16.75	**25.67**
80-84	15433	11871	**27304**	7.38	5.68	13.06	36050	51508	**87558**	42.81	23.05	**31.18**
85-89	9930	9601	**19531**	4.75	4.59	9.34	21131	34197	**55328**	46.99	28.08	**35.30**
90-94	4549	5738	**10287**	2.18	2.75	4.92	9147	17225	**26372**	49.73	33.31	**39.01**
95-99	1241	1880	**3121**	0.59	0.90	1.49	2571	5410	**7981**	48.27	34.75	**39.11**
100-104	124	206	**330**	0.06	0.10	0.16	374	665	**1039**	33.16	30.98	**31.76**
105-110	17	44	**61**	0.01	0.02	0.03	87	177	**264**	19.54	24.86	**23.11**
**Total**	**139013**	**70013**	**209026**	**66.51**	**33.49**		**2274349**	**2352834**	**4627183**	**6.11**	**2.98**	**4.52**

eTable 17. Obesity prevalence and proportions of patient population by age and sex

	Obesity (n)			Percent of obesity patients			Total population (n)			Prevalence (%)		
**Age group**	**Male**	**Female**	**Total**	**Male**	**Female**	**Total**	**Male**	**Female**	**Total**	**Male**	**Female**	**Total**
0-4	4429	4090	**8519**	0.52	0.48	0.99	248558	234757	**483315**	1.78	1.74	**1.76**
5-9	25234	22938	**48172**	2.93	2.67	5.60	231281	220106	**451387**	10.91	10.42	**10.67**
10-14	37105	32693	**69798**	4.31	3.80	8.12	199945	190186	**390131**	18.56	17.19	**17.89**
15-19	36548	31257	**67805**	4.25	3.63	7.88	163169	156268	**319437**	22.40	20.00	**21.23**
20-24	22055	20667	**42722**	2.56	2.40	4.97	132509	143680	**276189**	16.64	14.38	**15.47**
25-29	19236	19691	**38927**	2.24	2.29	4.53	156927	159023	**315950**	12.26	12.38	**12.32**
30-34	16281	20415	**36696**	1.89	2.37	4.27	162004	165350	**327354**	10.05	12.35	**11.21**
35-39	18145	22762	**40907**	2.11	2.65	4.76	156661	158737	**315398**	11.58	14.34	**12.97**
40-44	22150	25938	**48088**	2.58	3.02	5.59	145144	143572	**288716**	15.26	18.07	**16.66**
45-49	22840	26780	**49620**	2.66	3.11	5.77	115926	112939	**228865**	19.70	23.71	**21.68**
50-54	22103	28641	**50744**	2.57	3.33	5.90	93815	98655	**192470**	23.56	29.03	**26.36**
55-59	24808	34085	**58893**	2.88	3.96	6.85	91223	101690	**192913**	27.19	33.52	**30.53**
60-64	28275	38472	**66747**	3.29	4.47	7.76	93150	105570	**198720**	30.35	36.44	**33.59**
65-69	30039	40840	**70879**	3.49	4.75	8.24	93093	106207	**199300**	32.27	38.45	**35.56**
70-74	25427	35611	**61038**	2.96	4.14	7.10	76573	90083	**166656**	33.21	39.53	**36.63**
75-79	14606	23218	**37824**	1.70	2.70	4.40	45011	56829	**101840**	32.45	40.86	**37.14**
80-84	11172	21616	**32788**	1.30	2.51	3.81	36050	51508	**87558**	30.99	41.97	**37.45**
85-89	5930	13710	**19640**	0.69	1.59	2.28	21131	34197	**55328**	28.06	40.09	**35.50**
90-94	2117	5825	**7942**	0.25	0.68	0.92	9147	17225	**26372**	23.14	33.82	**30.12**
95-99	466	1492	**1958**	0.05	0.17	0.23	2571	5410	**7981**	18.13	27.58	**24.53**
100-104	62	146	**208**	0.01	0.02	0.02	374	665	**1039**	16.58	21.95	**20.02**
105-110	9	21	**30**	0.00	0.00	0.00	87	177	**264**	10.34	11.86	**11.36**
**Total**	**389037**	**470908**	**859945**	**45.24**	**54.76**		**2274349**	**2352834**	**4627183**	**17.11**	**20.01**	**18.58**

eTable 18. Osteoarthritis prevalence and proportions of patient population by age and sex

	Osteoarthritis (n)			Percent of osteoarthritis patients			Total population (n)			Prevalence (%)		
**Age group**	**Male**	**Female**	**Total**	**Male**	**Female**	**Total**	**Male**	**Female**	**Total**	**Male**	**Female**	**Total**
0-4	0	1	**1**	0.00	0.00	0.00	248558	234757	**483315**	0.00	0.00	**0.00**
5-9	4	0	**4**	0.00	0.00	0.00	231281	220106	**451387**	0.00	0.00	**0.00**
10-14	5	3	**8**	0.00	0.00	0.01	199945	190186	**390131**	0.00	0.00	**0.00**
15-19	3	7	**10**	0.00	0.01	0.01	163169	156268	**319437**	0.00	0.00	**0.00**
20-24	28	14	**42**	0.03	0.01	0.04	132509	143680	**276189**	0.02	0.01	**0.02**
25-29	88	38	**126**	0.09	0.04	0.13	156927	159023	**315950**	0.06	0.02	**0.04**
30-34	122	92	**214**	0.12	0.09	0.21	162004	165350	**327354**	0.08	0.06	**0.07**
35-39	208	198	**406**	0.21	0.20	0.41	156661	158737	**315398**	0.13	0.12	**0.13**
40-44	375	418	**793**	0.37	0.42	0.79	145144	143572	**288716**	0.26	0.29	**0.27**
45-49	564	1060	**1624**	0.56	1.06	1.62	115926	112939	**228865**	0.49	0.94	**0.71**
50-54	893	2265	**3158**	0.89	2.26	3.15	93815	98655	**192470**	0.95	2.30	**1.64**
55-59	1609	4588	**6197**	1.61	4.58	6.19	91223	101690	**192913**	1.76	4.51	**3.21**
60-64	2677	7623	**10300**	2.67	7.61	10.29	93150	105570	**198720**	2.87	7.22	**5.18**
65-69	4073	10629	**14702**	4.07	10.62	14.69	93093	106207	**199300**	4.38	10.01	**7.38**
70-74	4348	11883	**16231**	4.34	11.87	16.21	76573	90083	**166656**	5.68	13.19	**9.74**
75-79	3556	10226	**13782**	3.55	10.21	13.77	45011	56829	**101840**	7.90	17.99	**13.53**
80-84	3533	11264	**14797**	3.53	11.25	14.78	36050	51508	**87558**	9.80	21.87	**16.90**
85-89	2550	8344	**10894**	2.55	8.33	10.88	21131	34197	**55328**	12.07	24.40	**19.69**
90-94	1171	3981	**5152**	1.17	3.98	5.15	9147	17225	**26372**	12.80	23.11	**19.54**
95-99	309	1158	**1467**	0.31	1.16	1.47	2571	5410	**7981**	12.02	21.40	**18.38**
100-104	56	124	**180**	0.06	0.12	0.18	374	665	**1039**	14.97	18.65	**17.32**
105-110	6	19	**25**	0.01	0.02	0.02	87	177	**264**	6.90	10.73	**9.47**
**Total**	**26178**	**73935**	**100113**	**26.15**	**73.85**		**2274349**	**2352834**	**4627183**	**1.15**	**3.14**	**2.16**

eTable 19. Osteoporosis prevalence and proportions of patient population by age and sex

	Osteoporosis (n)			Percent of osteoporosis patients			Total population (n)			Prevalence (%)		
**Age group**	**Male**	**Female**	**Total**	**Male**	**Female**	**Total**	**Male**	**Female**	**Total**	**Male**	**Female**	**Total**
0-4	9	9	**18**	0.01	0.01	0.01	248558	234757	**483315**	0.00	0.00	**0.00**
5-9	37	21	**58**	0.02	0.01	0.03	231281	220106	**451387**	0.02	0.01	**0.01**
10-14	48	41	**89**	0.03	0.02	0.05	199945	190186	**390131**	0.02	0.02	**0.02**
15-19	76	63	**139**	0.04	0.04	0.08	163169	156268	**319437**	0.05	0.04	**0.04**
20-24	130	84	**214**	0.07	0.05	0.12	132509	143680	**276189**	0.10	0.06	**0.08**
25-29	188	148	**336**	0.11	0.08	0.19	156927	159023	**315950**	0.12	0.09	**0.11**
30-34	275	290	**565**	0.16	0.16	0.32	162004	165350	**327354**	0.17	0.18	**0.17**
35-39	327	405	**732**	0.19	0.23	0.42	156661	158737	**315398**	0.21	0.26	**0.23**
40-44	440	581	**1021**	0.25	0.33	0.58	145144	143572	**288716**	0.30	0.40	**0.35**
45-49	474	815	**1289**	0.27	0.46	0.73	115926	112939	**228865**	0.41	0.72	**0.56**
50-54	496	2380	**2876**	0.28	1.35	1.63	93815	98655	**192470**	0.53	2.41	**1.49**
55-59	842	6411	**7253**	0.48	3.64	4.12	91223	101690	**192913**	0.92	6.30	**3.76**
60-64	1414	14287	**15701**	0.80	8.12	8.92	93150	105570	**198720**	1.52	13.53	**7.90**
65-69	2246	23650	**25896**	1.28	13.44	14.72	93093	106207	**199300**	2.41	22.27	**12.99**
70-74	3067	27342	**30409**	1.74	15.54	17.29	76573	90083	**166656**	4.01	30.35	**18.25**
75-79	2885	22673	**25558**	1.64	12.89	14.53	45011	56829	**101840**	6.41	39.90	**25.10**
80-84	3386	24360	**27746**	1.92	13.85	15.77	36050	51508	**87558**	9.39	47.29	**31.69**
85-89	2545	18608	**21153**	1.45	10.58	12.02	21131	34197	**55328**	12.04	54.41	**38.23**
90-94	1342	9716	**11058**	0.76	5.52	6.29	9147	17225	**26372**	14.67	56.41	**41.93**
95-99	355	3018	**3373**	0.20	1.72	1.92	2571	5410	**7981**	13.81	55.79	**42.26**
100-104	52	327	**379**	0.03	0.19	0.22	374	665	**1039**	13.90	49.17	**36.48**
105-110	6	55	**61**	0.00	0.03	0.03	87	177	**264**	6.90	31.07	**23.11**
**Total**	**20640**	**155284**	**175924**	**11.73**	**88.27**		**2274349**	**2352834**	**4627183**	**0.91**	**6.60**	**3.80**

eTable 20. Parkinson's disease prevalence and proportions of patient population by age and sex

	Parkinson's disease (n)			Percent of Parkinson's disease patients			Total population (n)			Prevalence (%)		
**Age group**	**Male**	**Female**	**Total**	**Male**	**Female**	**Total**	**Male**	**Female**	**Total**	**Male**	**Female**	**Total**
0-4	1	1	**2**	0.01	0.01	0.01	248558	234757	**483315**	0.00	0.00	**0.00**
5-9	1	2	**3**	0.01	0.01	0.02	231281	220106	**451387**	0.00	0.00	**0.00**
10-14	2	1	**3**	0.01	0.01	0.02	199945	190186	**390131**	0.00	0.00	**0.00**
15-19	4	4	**8**	0.02	0.02	0.04	163169	156268	**319437**	0.00	0.00	**0.00**
20-24	7	5	**12**	0.04	0.03	0.07	132509	143680	**276189**	0.01	0.00	**0.00**
25-29	16	12	**28**	0.09	0.07	0.16	156927	159023	**315950**	0.01	0.01	**0.01**
30-34	22	12	**34**	0.12	0.07	0.19	162004	165350	**327354**	0.01	0.01	**0.01**
35-39	63	38	**101**	0.35	0.21	0.57	156661	158737	**315398**	0.04	0.02	**0.03**
40-44	132	104	**236**	0.74	0.58	1.32	145144	143572	**288716**	0.09	0.07	**0.08**
45-49	236	201	**437**	1.32	1.13	2.45	115926	112939	**228865**	0.20	0.18	**0.19**
50-54	341	277	**618**	1.91	1.55	3.47	93815	98655	**192470**	0.36	0.28	**0.32**
55-59	510	400	**910**	2.86	2.24	5.10	91223	101690	**192913**	0.56	0.39	**0.47**
60-64	723	568	**1291**	4.05	3.19	7.24	93150	105570	**198720**	0.78	0.54	**0.65**
65-69	1165	887	**2052**	6.53	4.97	11.51	93093	106207	**199300**	1.25	0.84	**1.03**
70-74	1443	1159	**2602**	8.09	6.50	14.59	76573	90083	**166656**	1.88	1.29	**1.56**
75-79	1360	1214	**2574**	7.63	6.81	14.44	45011	56829	**101840**	3.02	2.14	**2.53**
80-84	1430	1567	**2997**	8.02	8.79	16.81	36050	51508	**87558**	3.97	3.04	**3.42**
85-89	1094	1283	**2377**	6.14	7.20	13.33	21131	34197	**55328**	5.18	3.75	**4.30**
90-94	473	699	**1172**	2.65	3.92	6.57	9147	17225	**26372**	5.17	4.06	**4.44**
95-99	116	224	**340**	0.65	1.26	1.91	2571	5410	**7981**	4.51	4.14	**4.26**
100-104	15	16	**31**	0.08	0.09	0.17	374	665	**1039**	4.01	2.41	**2.98**
105-110	1	1	**2**	0.01	0.01	0.01	87	177	**264**	1.15	0.56	**0.76**
**Total**	**9155**	**8675**	**17830**	**51.35**	**48.65**		**2274349**	**2352834**	**4627183**	**0.40**	**0.37**	**0.39**

eTable 21. Peripheral vascular disease (including aortic disease) prevalence and proportions of patient population by age and sex

	Peripheral artery diesease, aortic aneurism (n)			Percent of peripheral artery diesease, aortic aneurism patients			Total population (n)			Prevalence (%)		
**Age group**	**Male**	**Female**	**Total**	**Male**	**Female**	**Total**	**Male**	**Female**	**Total**	**Male**	**Female**	**Total**
0-4	7	8	**15**	0.01	0.01	0.02	248558	234757	**483315**	0.00	0.00	**0.00**
5-9	13	7	**20**	0.02	0.01	0.03	231281	220106	**451387**	0.01	0.00	**0.00**
10-14	23	21	**44**	0.03	0.03	0.06	199945	190186	**390131**	0.01	0.01	**0.01**
15-19	72	53	**125**	0.09	0.07	0.16	163169	156268	**319437**	0.04	0.03	**0.04**
20-24	98	39	**137**	0.12	0.05	0.17	132509	143680	**276189**	0.07	0.03	**0.05**
25-29	123	93	**216**	0.15	0.12	0.27	156927	159023	**315950**	0.08	0.06	**0.07**
30-34	139	123	**262**	0.17	0.15	0.33	162004	165350	**327354**	0.09	0.07	**0.08**
35-39	237	193	**430**	0.30	0.24	0.54	156661	158737	**315398**	0.15	0.12	**0.14**
40-44	402	277	**679**	0.50	0.35	0.85	145144	143572	**288716**	0.28	0.19	**0.24**
45-49	662	369	**1031**	0.83	0.46	1.29	115926	112939	**228865**	0.57	0.33	**0.45**
50-54	1167	686	**1853**	1.46	0.86	2.32	93815	98655	**192470**	1.24	0.70	**0.96**
55-59	2471	1271	**3742**	3.10	1.59	4.69	91223	101690	**192913**	2.71	1.25	**1.94**
60-64	4634	2477	**7111**	5.81	3.11	8.92	93150	105570	**198720**	4.97	2.35	**3.58**
65-69	7216	4019	**11235**	9.05	5.04	14.09	93093	106207	**199300**	7.75	3.78	**5.64**
70-74	8453	5368	**13821**	10.60	6.73	17.33	76573	90083	**166656**	11.04	5.96	**8.29**
75-79	6681	4905	**11586**	8.38	6.15	14.53	45011	56829	**101840**	14.84	8.63	**11.38**
80-84	6691	5904	**12595**	8.39	7.40	15.80	36050	51508	**87558**	18.56	11.46	**14.38**
85-89	4556	4539	**9095**	5.71	5.69	11.41	21131	34197	**55328**	21.56	13.27	**16.44**
90-94	2081	2282	**4363**	2.61	2.86	5.47	9147	17225	**26372**	22.75	13.25	**16.54**
95-99	504	708	**1212**	0.63	0.89	1.52	2571	5410	**7981**	19.60	13.09	**15.19**
100-104	55	88	**143**	0.07	0.11	0.18	374	665	**1039**	14.71	13.23	**13.76**
105-110	7	11	**18**	0.01	0.01	0.02	87	177	**264**	8.05	6.21	**6.82**
**Total**	**46292**	**33441**	**79733**	**58.06**	**41.94**		**2274349**	**2352834**	**4627183**	**2.04**	**1.42**	**1.72**

eTable 22. Benign prostatic hypertrophy prevalence in men and proportions of patient population by age and sex

	Prostatic hypertrophy (n)	Percent of prostatic hypertrophy patients	Total population (n)	Prevalence (%)
Age group	Male	Male	Male	Male
20-24	2	0.00	132509	0.00
25-29	9	0.01	156927	0.01
30-34	27	0.03	162004	0.02
35-39	39	0.04	156661	0.02
40-44	113	0.13	145144	0.08
45-49	338	0.38	115926	0.29
50-54	964	1.08	93815	1.03
55-59	2747	3.07	91223	3.01
60-64	6913	7.72	93150	7.42
65-69	13426	15.00	93093	14.42
70-74	18640	20.82	76573	24.34
75-79	14649	16.36	45011	32.55
80-84	15397	17.20	36050	42.71
85-89	10208	11.40	21131	48.31
90-94	4629	5.17	9147	50.61
95-99	1256	1.40	2571	48.85
100-104	142	0.16	374	37.97
105-110	19	0.02	87	21.84
**Total**	**89518**		**1431396**	3.94

eTable 23. Chronic renal failure prevalence and proportions of patient population by age and sex

	Chronic renal failure (n)			Percent of chronic renal failure patients			Total population (n)			Prevalence (%)		
**Age group**	**Male**	**Female**	**Total**	**Male**	**Female**	**Total**	**Male**	**Female**	**Total**	**Male**	**Female**	**Total**
0-4	116	73	**189**	0.14	0.09	0.22	248558	234757	**483315**	0.05	0.03	**0.04**
5-9	137	99	**236**	0.16	0.12	0.28	231281	220106	**451387**	0.06	0.04	**0.05**
10-14	146	87	**233**	0.17	0.10	0.28	199945	190186	**390131**	0.07	0.05	**0.06**
15-19	196	148	**344**	0.23	0.18	0.41	163169	156268	**319437**	0.12	0.09	**0.11**
20-24	278	189	**467**	0.33	0.22	0.55	132509	143680	**276189**	0.21	0.13	**0.17**
25-29	398	214	**612**	0.47	0.25	0.73	156927	159023	**315950**	0.25	0.13	**0.19**
30-34	603	277	**880**	0.72	0.33	1.05	162004	165350	**327354**	0.37	0.17	**0.27**
35-39	761	355	**1116**	0.90	0.42	1.33	156661	158737	**315398**	0.49	0.22	**0.35**
40-44	1015	413	**1428**	1.21	0.49	1.70	145144	143572	**288716**	0.70	0.29	**0.49**
45-49	1249	495	**1744**	1.48	0.59	2.07	115926	112939	**228865**	1.08	0.44	**0.76**
50-54	1650	744	**2394**	1.96	0.88	2.84	93815	98655	**192470**	1.76	0.75	**1.24**
55-59	2508	1065	**3573**	2.98	1.27	4.24	91223	101690	**192913**	2.75	1.05	**1.85**
60-64	4213	1770	**5983**	5.00	2.10	7.11	93150	105570	**198720**	4.52	1.68	**3.01**
65-69	6687	3026	**9713**	7.94	3.59	11.54	93093	106207	**199300**	7.18	2.85	**4.87**
70-74	8408	4121	**12529**	9.99	4.90	14.88	76573	90083	**166656**	10.98	4.57	**7.52**
75-79	7047	4099	**11146**	8.37	4.87	13.24	45011	56829	**101840**	15.66	7.21	**10.94**
80-84	7891	5744	**13635**	9.37	6.82	16.20	36050	51508	**87558**	21.89	11.15	**15.57**
85-89	5642	4782	**10424**	6.70	5.68	12.38	21131	34197	**55328**	26.70	13.98	**18.84**
90-94	2802	2797	**5599**	3.33	3.32	6.65	9147	17225	**26372**	30.63	16.24	**21.23**
95-99	789	903	**1692**	0.94	1.07	2.01	2571	5410	**7981**	30.69	16.69	**21.20**
100-104	111	96	**207**	0.13	0.11	0.25	374	665	**1039**	29.68	14.44	**19.92**
105-110	14	19	**33**	0.02	0.02	0.04	87	177	**264**	16.09	10.73	**12.50**
**Total**	**52661**	**31516**	**84177**	**62.56**	**37.44**		**2274349**	**2352834**	**4627183**	**2.32**	**1.34**	**1.82**

eTable 24. Rheumatoid arthritis prevalence and proportions of patient population by age and sex

	Rheumatoid arthritis (n)			Percent of rheumatoid arthritis patients			Total population (n)			Prevalence (%)		
**Age group**	**Male**	**Female**	**Total**	**Male**	**Female**	**Total**	**Male**	**Female**	**Total**	**Male**	**Female**	**Total**
0-4	11	54	**65**	0.05	0.24	0.29	248558	234757	**483315**	0.00	0.02	**0.01**
5-9	58	121	**179**	0.26	0.54	0.80	231281	220106	**451387**	0.03	0.05	**0.04**
10-14	85	154	**239**	0.38	0.69	1.07	199945	190186	**390131**	0.04	0.08	**0.06**
15-19	103	190	**293**	0.46	0.85	1.31	163169	156268	**319437**	0.06	0.12	**0.09**
20-24	91	220	**311**	0.41	0.98	1.39	132509	143680	**276189**	0.07	0.15	**0.11**
25-29	130	315	**445**	0.58	1.41	1.99	156927	159023	**315950**	0.08	0.20	**0.14**
30-34	152	438	**590**	0.68	1.96	2.64	162004	165350	**327354**	0.09	0.26	**0.18**
35-39	165	550	**715**	0.74	2.46	3.20	156661	158737	**315398**	0.11	0.35	**0.23**
40-44	246	728	**974**	1.10	3.26	4.36	145144	143572	**288716**	0.17	0.51	**0.34**
45-49	271	836	**1107**	1.21	3.74	4.95	115926	112939	**228865**	0.23	0.74	**0.48**
50-54	362	1056	**1418**	1.62	4.72	6.34	93815	98655	**192470**	0.39	1.07	**0.74**
55-59	456	1453	**1909**	2.04	6.50	8.54	91223	101690	**192913**	0.50	1.43	**0.99**
60-64	580	1924	**2504**	2.59	8.60	11.20	93150	105570	**198720**	0.62	1.82	**1.26**
65-69	658	2124	**2782**	2.94	9.50	12.44	93093	106207	**199300**	0.71	2.00	**1.40**
70-74	687	2108	**2795**	3.07	9.43	12.50	76573	90083	**166656**	0.90	2.34	**1.68**
75-79	465	1598	**2063**	2.08	7.15	9.23	45011	56829	**101840**	1.03	2.81	**2.03**
80-84	393	1533	**1926**	1.76	6.86	8.61	36050	51508	**87558**	1.09	2.98	**2.20**
85-89	265	1003	**1268**	1.19	4.49	5.67	21131	34197	**55328**	1.25	2.93	**2.29**
90-94	116	487	**603**	0.52	2.18	2.70	9147	17225	**26372**	1.27	2.83	**2.29**
95-99	33	124	**157**	0.15	0.55	0.70	2571	5410	**7981**	1.28	2.29	**1.97**
100-104	3	14	**17**	0.01	0.06	0.08	374	665	**1039**	0.80	2.11	**1.64**
105-110	2	0	**2**	0.01	0.00	0.01	87	177	**264**	2.30	0.00	**0.76**
**Total**	**5332**	**17030**	**22362**	**23.84**	**76.16**		**2274349**	**2352834**	**4627183**	**0.23**	**0.72**	**0.48**

eTable 25. Schizophrenia prevalence and proportions of patient population by age and sex

	Schizophrenia (n)			Percent of schizophrenia patients			Total population (n)			Prevalence (%)		
**Age group**	**Male**	**Female**	**Total**	**Male**	**Female**	**Total**	**Male**	**Female**	**Total**	**Male**	**Female**	**Total**
0-4	3	2	**5**	0.01	0.01	0.02	248558	234757	**483315**	0.00	0.00	**0.00**
5-9	13	4	**17**	0.04	0.01	0.05	231281	220106	**451387**	0.01	0.00	**0.00**
10-14	37	24	**61**	0.11	0.07	0.18	199945	190186	**390131**	0.02	0.01	**0.02**
15-19	251	119	**370**	0.76	0.36	1.12	163169	156268	**319437**	0.15	0.08	**0.12**
20-24	877	358	**1235**	2.65	1.08	3.73	132509	143680	**276189**	0.66	0.25	**0.45**
25-29	1665	587	**2252**	5.03	1.77	6.81	156927	159023	**315950**	1.06	0.37	**0.71**
30-34	2037	845	**2882**	6.16	2.55	8.71	162004	165350	**327354**	1.26	0.51	**0.88**
35-39	2238	1111	**3349**	6.76	3.36	10.12	156661	158737	**315398**	1.43	0.70	**1.06**
40-44	2219	1172	**3391**	6.71	3.54	10.25	145144	143572	**288716**	1.53	0.82	**1.17**
45-49	2100	1286	**3386**	6.35	3.89	10.23	115926	112939	**228865**	1.81	1.14	**1.48**
50-54	1872	1261	**3133**	5.66	3.81	9.47	93815	98655	**192470**	2.00	1.28	**1.63**
55-59	1832	1415	**3247**	5.54	4.28	9.81	91223	101690	**192913**	2.01	1.39	**1.68**
60-64	1703	1473	**3176**	5.15	4.45	9.60	93150	105570	**198720**	1.83	1.40	**1.60**
65-69	1339	1353	**2692**	4.05	4.09	8.14	93093	106207	**199300**	1.44	1.27	**1.35**
70-74	854	950	**1804**	2.58	2.87	5.45	76573	90083	**166656**	1.12	1.05	**1.08**
75-79	353	561	**914**	1.07	1.70	2.76	45011	56829	**101840**	0.78	0.99	**0.90**
80-84	210	490	**700**	0.63	1.48	2.12	36050	51508	**87558**	0.58	0.95	**0.80**
85-89	92	224	**316**	0.28	0.68	0.96	21131	34197	**55328**	0.44	0.66	**0.57**
90-94	26	101	**127**	0.08	0.31	0.38	9147	17225	**26372**	0.28	0.59	**0.48**
95-99	7	21	**28**	0.02	0.06	0.08	2571	5410	**7981**	0.27	0.39	**0.35**
100-104	1	1	**2**	0.00	0.00	0.01	374	665	**1039**	0.27	0.15	**0.19**
105-110	0	0	**0**	0.00	0.00	0.00	87	177	**264**	0.00	0.00	**0.00**
**Total**	**19729**	**13358**	**33087**	**59.63**	**40.37**		**2274349**	**2352834**	**4627183**	**0.87**	**0.57**	**0.72**

eTable 26. Thyroid disease prevalence and proportions of patient population by age and sex

	Thyroid disease (n)			Percent of thyroid disease patients			Total population (n)			Prevalence (%)		
Age group	Male	Female	Total	Male	Female	Total	Male	Female	Total	Male	Female	Total
0-4	408	472	**880**	0.17	0.19	0.36	248558	234757	**483315**	0.16	0.20	**0.18**
5-9	603	568	**1171**	0.25	0.23	0.48	231281	220106	**451387**	0.26	0.26	**0.26**
10-14	842	945	**1787**	0.34	0.38	0.73	199945	190186	**390131**	0.42	0.50	**0.46**
15-19	1175	1860	**3035**	0.48	0.76	1.24	163169	156268	**319437**	0.72	1.19	**0.95**
20-24	1114	3866	**4980**	0.45	1.57	2.03	132509	143680	**276189**	0.84	2.69	**1.80**
25-29	1555	8129	**9684**	0.63	3.31	3.94	156927	159023	**315950**	0.99	5.11	**3.07**
30-34	2121	12182	**14303**	0.86	4.96	5.82	162004	165350	**327354**	1.31	7.37	**4.37**
35-39	2650	13744	**16394**	1.08	5.59	6.67	156661	158737	**315398**	1.69	8.66	**5.20**
40-44	2791	13429	**16220**	1.14	5.47	6.60	145144	143572	**288716**	1.92	9.35	**5.62**
45-49	2834	11771	**14605**	1.15	4.79	5.94	115926	112939	**228865**	2.44	10.42	**6.38**
50-54	2873	11486	**14359**	1.17	4.68	5.84	93815	98655	**192470**	3.06	11.64	**7.46**
55-59	3375	13961	**17336**	1.37	5.68	7.06	91223	101690	**192913**	3.70	13.73	**8.99**
60-64	4354	17626	**21980**	1.77	7.17	8.95	93150	105570	**198720**	4.67	16.70	**11.06**
65-69	5467	21080	**26547**	2.23	8.58	10.81	93093	106207	**199300**	5.87	19.85	**13.32**
70-74	6204	20570	**26774**	2.53	8.37	10.90	76573	90083	**166656**	8.10	22.83	**16.07**
75-79	4243	13500	**17743**	1.73	5.50	7.22	45011	56829	**101840**	9.43	23.76	**17.42**
80-84	4248	13091	**17339**	1.73	5.33	7.06	36050	51508	**87558**	11.78	25.42	**19.80**
85-89	2876	9182	**12058**	1.17	3.74	4.91	21131	34197	**55328**	13.61	26.85	**21.79**
90-94	1337	4956	**6293**	0.54	2.02	2.56	9147	17225	**26372**	14.62	28.77	**23.86**
95-99	453	1520	**1973**	0.18	0.62	0.80	2571	5410	**7981**	17.62	28.10	**24.72**
100-104	39	146	**185**	0.02	0.06	0.08	374	665	**1039**	10.43	21.95	**17.81**
105-110	7	23	**30**	0.00	0.01	0.01	87	177	**264**	8.05	12.99	**11.36**
**Total**	**51569**	**194107**	**245676**	**20.99**	**79.01**		**2274349**	**2352834**	**4627183**	**2.27**	**8.25**	**5.31**

eTable 27. Bladder cancer prevalence in adults and proportions of patient population by age and sex

	Bladder cancer (n)			Percent of adult bladder cancer patients			Total population (n)			Prevalence (%)		
Age group	Male	Female	Total	Male	Female	Total	Male	Female	Total	Male	Female	Total
20-24	1	0	**1**	0.03	0.00	0.03	132509	143680	**276189**	0.00	0.00	**0.00**
25-29	2	3	**5**	0.06	0.09	0.15	156927	159023	**315950**	0.00	0.00	**0.00**
30-34	8	4	**12**	0.24	0.12	0.36	162004	165350	**327354**	0.00	0.00	**0.00**
35-39	17	8	**25**	0.52	0.24	0.76	156661	158737	**315398**	0.01	0.01	**0.01**
40-44	31	16	**47**	0.94	0.49	1.43	145144	143572	**288716**	0.02	0.01	**0.02**
45-49	41	10	**51**	1.25	0.30	1.55	115926	112939	**228865**	0.04	0.01	**0.02**
50-54	59	15	**74**	1.79	0.46	2.25	93815	98655	**192470**	0.06	0.02	**0.04**
55-59	128	36	**164**	3.89	1.09	4.99	91223	101690	**192913**	0.14	0.04	**0.09**
60-64	231	61	**292**	7.02	1.85	8.88	93150	105570	**198720**	0.25	0.06	**0.15**
65-69	448	90	**538**	13.62	2.74	16.36	93093	106207	**199300**	0.48	0.08	**0.27**
70-74	518	117	**635**	15.75	3.56	19.31	76573	90083	**166656**	0.68	0.13	**0.38**
75-79	368	98	**466**	11.19	2.98	14.17	45011	56829	**101840**	0.82	0.17	**0.46**
80-84	403	104	**507**	12.25	3.16	15.42	36050	51508	**87558**	1.12	0.20	**0.58**
85-89	267	55	**322**	8.12	1.67	9.79	21131	34197	**55328**	1.26	0.16	**0.58**
90-94	84	33	**117**	2.55	1.00	3.56	9147	17225	**26372**	0.92	0.19	**0.44**
95-99	24	7	**31**	0.73	0.21	0.94	2571	5410	**7981**	0.93	0.13	**0.39**
100-104	2	0	**2**	0.06	0.00	0.06	374	665	**1039**	0.53	0.00	**0.19**
105-110	0	0	**0**	0.00	0.00	0.00	87	177	**264**	0.00	0.00	**0.00**
**Total**	**2632**	**657**	**3289**	**80.02**	**19.98**		**1431396**	**1551517**	**2982913**	**0.18**	**0.04**	**0.11**

eTable 28. Bone cancer prevalence in adults and proportions of patient population by age and sex

	Malignancy of bone (n)			Percent of adult bone cancer patients			Total population (n)			Prevalence (%)		
**Age group**	**Male**	**Female**	**Total**	**Male**	**Female**	**Total**	**Male**	**Female**	**Total**	**Male**	**Female**	**Total**
20-24	15	7	**22**	4.10	1.91	6.01	132509	143680	**276189**	0.01	0.00	**0.01**
25-29	5	6	**11**	1.37	1.64	3.01	156927	159023	**315950**	0.00	0.00	**0.00**
30-34	11	13	**24**	3.01	3.55	6.56	162004	165350	**327354**	0.01	0.01	**0.01**
35-39	8	11	**19**	2.19	3.01	5.19	156661	158737	**315398**	0.01	0.01	**0.01**
40-44	9	19	**28**	2.46	5.19	7.65	145144	143572	**288716**	0.01	0.01	**0.01**
45-49	4	11	**15**	1.09	3.01	4.10	115926	112939	**228865**	0.00	0.01	**0.01**
50-54	10	13	**23**	2.73	3.55	6.28	93815	98655	**192470**	0.01	0.01	**0.01**
55-59	9	10	**19**	2.46	2.73	5.19	91223	101690	**192913**	0.01	0.01	**0.01**
60-64	16	21	**37**	4.37	5.74	10.11	93150	105570	**198720**	0.02	0.02	**0.02**
65-69	23	18	**41**	6.28	4.92	11.20	93093	106207	**199300**	0.02	0.02	**0.02**
70-74	24	21	**45**	6.56	5.74	12.30	76573	90083	**166656**	0.03	0.02	**0.03**
75-79	20	14	**34**	5.46	3.83	9.29	45011	56829	**101840**	0.04	0.02	**0.03**
80-84	12	11	**23**	3.28	3.01	6.28	36050	51508	**87558**	0.03	0.02	**0.03**
85-89	8	7	**15**	2.19	1.91	4.10	21131	34197	**55328**	0.04	0.02	**0.03**
90-94	4	4	**8**	1.09	1.09	2.19	9147	17225	**26372**	0.04	0.02	**0.03**
95-99	1	1	**2**	0.27	0.27	0.55	2571	5410	**7981**	0.04	0.02	**0.03**
100-104	0	0	**0**	0.00	0.00	0.00	374	665	**1039**	0.00	0.00	**0.00**
105-110	0	0	**0**	0.00	0.00	0.00	87	177	**264**	0.00	0.00	**0.00**
**Total**	**179**	**187**	**366**	**48.91**	**51.09**		**1431396**	**1551517**	**2982913**	**0.01**	**0.01**	**0.01**

eTable 29. Brain/central nervous system (CNS) cancer prevalence in adults and proportions of patient population by age and sex

	Malignancy of brain/CNS (n)			Percent of adult brain/CNS cancer patients			Total population (n)			Prevalence (%)		
**Age group**	**Male**	**Female**	**Total**	**Male**	**Female**	**Total**	**Male**	**Female**	**Total**	**Male**	**Female**	**Total**
20-24	13	4	**17**	1.56	0.48	2.04	132509	143680	**276189**	0.01	0.00	**0.01**
25-29	9	18	**27**	1.08	2.16	3.24	156927	159023	**315950**	0.01	0.01	**0.01**
30-34	19	17	**36**	2.28	2.04	4.32	162004	165350	**327354**	0.01	0.01	**0.01**
35-39	29	22	**51**	3.48	2.64	6.12	156661	158737	**315398**	0.02	0.01	**0.02**
40-44	38	33	**71**	4.56	3.96	8.52	145144	143572	**288716**	0.03	0.02	**0.02**
45-49	33	25	**58**	3.96	3.00	6.96	115926	112939	**228865**	0.03	0.02	**0.03**
50-54	15	29	**44**	1.80	3.48	5.28	93815	98655	**192470**	0.02	0.03	**0.02**
55-59	30	36	**66**	3.60	4.32	7.92	91223	101690	**192913**	0.03	0.04	**0.03**
60-64	51	41	**92**	6.12	4.92	11.04	93150	105570	**198720**	0.05	0.04	**0.05**
65-69	61	56	**117**	7.32	6.72	14.05	93093	106207	**199300**	0.07	0.05	**0.06**
70-74	56	65	**121**	6.72	7.80	14.53	76573	90083	**166656**	0.07	0.07	**0.07**
75-79	26	42	**68**	3.12	5.04	8.16	45011	56829	**101840**	0.06	0.07	**0.07**
80-84	21	23	**44**	2.52	2.76	5.28	36050	51508	**87558**	0.06	0.04	**0.05**
85-89	6	13	**19**	0.72	1.56	2.28	21131	34197	**55328**	0.03	0.04	**0.03**
90-94	0	2	**2**	0.00	0.24	0.24	9147	17225	**26372**	0.00	0.01	**0.01**
95-99	0	0	**0**	0.00	0.00	0.00	2571	5410	**7981**	0.00	0.00	**0.00**
100-104	0	0	**0**	0.00	0.00	0.00	374	665	**1039**	0.00	0.00	**0.00**
105-110	0	0	**0**	0.00	0.00	0.00	87	177	**264**	0.00	0.00	**0.00**
**Total**	**407**	**426**	**833**	**48.86**	**51.14**		**1431396**	**1551517**	**2982913**	**0.03**	**0.03**	**0.03**

eTable 30. Breast cancer prevalence in adults and proportions of patient population by age and sex

	Breast cancer (n)			Percent of adult breast cancer patients			Total population (n)			Prevalence (%)		
Age group	Male	Female	Total	Male	Female	Total	Male	Female	Total	Male	Female	Total
20-24	6	6	**12**	0.03	0.03	0.06	132509	143680	**276189**	0.00	0.00	**0.00**
25-29	5	54	**59**	0.02	0.26	0.28	156927	159023	**315950**	0.00	0.03	**0.02**
30-34	13	201	**214**	0.06	0.95	1.01	162004	165350	**327354**	0.01	0.12	**0.07**
35-39	8	508	**516**	0.04	2.40	2.44	156661	158737	**315398**	0.01	0.32	**0.16**
40-44	8	898	**906**	0.04	4.24	4.28	145144	143572	**288716**	0.01	0.63	**0.31**
45-49	7	1196	**1203**	0.03	5.65	5.68	115926	112939	**228865**	0.01	1.06	**0.53**
50-54	11	1682	**1693**	0.05	7.95	8.00	93815	98655	**192470**	0.01	1.70	**0.88**
55-59	12	2047	**2059**	0.06	9.67	9.73	91223	101690	**192913**	0.01	2.01	**1.07**
60-64	26	2479	**2505**	0.12	11.71	11.83	93150	105570	**198720**	0.03	2.35	**1.26**
65-69	32	3273	**3305**	0.15	15.46	15.61	93093	106207	**199300**	0.03	3.08	**1.66**
70-74	47	3389	**3436**	0.22	16.01	16.23	76573	90083	**166656**	0.06	3.76	**2.06**
75-79	34	2033	**2067**	0.16	9.60	9.76	45011	56829	**101840**	0.08	3.58	**2.03**
80-84	34	1678	**1712**	0.16	7.93	8.09	36050	51508	**87558**	0.09	3.26	**1.96**
85-89	26	920	**946**	0.12	4.35	4.47	21131	34197	**55328**	0.12	2.69	**1.71**
90-94	10	405	**415**	0.05	1.91	1.96	9147	17225	**26372**	0.11	2.35	**1.57**
95-99	2	109	**111**	0.01	0.51	0.52	2571	5410	**7981**	0.08	2.01	**1.39**
100-104	0	10	**10**	0.00	0.05	0.05	374	665	**1039**	0.00	1.50	**0.96**
105-110	0	1	**1**	0.00	0.00	0.00	87	177	**264**	0.00	0.56	**0.38**
**Total**	**281**	**20889**	**21170**	**1.33**	**98.67**		**1431396**	**1551517**	**2982913**	**0.02**	**1.35**	**0.71**

eTable 31. Colorectal cancer prevalence in adults and proportions of patient population by age and sex

	Malignancy of colon or rectum (n)			Percent of adult colorectal cancer patients			Total population (n)			Prevalence (%)		
**Age group**	**Male**	**Female**	**Total**	**Male**	**Female**	**Total**	**Male**	**Female**	**Total**	**Male**	**Female**	**Total**
20-24	5	3	**8**	0.09	0.06	0.15	132509	143680	**276189**	0.00	0.00	**0.00**
25-29	5	14	**19**	0.09	0.26	0.35	156927	159023	**315950**	0.00	0.01	**0.01**
30-34	20	20	**40**	0.37	0.37	0.74	162004	165350	**327354**	0.01	0.01	**0.01**
35-39	44	34	**78**	0.81	0.63	1.43	156661	158737	**315398**	0.03	0.02	**0.02**
40-44	65	64	**129**	1.20	1.18	2.37	145144	143572	**288716**	0.04	0.04	**0.04**
45-49	78	81	**159**	1.43	1.49	2.92	115926	112939	**228865**	0.07	0.07	**0.07**
50-54	119	139	**258**	2.19	2.56	4.75	93815	98655	**192470**	0.13	0.14	**0.13**
55-59	185	211	**396**	3.40	3.88	7.28	91223	101690	**192913**	0.20	0.21	**0.21**
60-64	281	279	**560**	5.17	5.13	10.30	93150	105570	**198720**	0.30	0.26	**0.28**
65-69	426	391	**817**	7.84	7.19	15.03	93093	106207	**199300**	0.46	0.37	**0.41**
70-74	480	450	**930**	8.83	8.28	17.11	76573	90083	**166656**	0.63	0.50	**0.56**
75-79	334	327	**661**	6.14	6.02	12.16	45011	56829	**101840**	0.74	0.58	**0.65**
80-84	364	336	**700**	6.70	6.18	12.88	36050	51508	**87558**	1.01	0.65	**0.80**
85-89	207	227	**434**	3.81	4.18	7.98	21131	34197	**55328**	0.98	0.66	**0.78**
90-94	105	94	**199**	1.93	1.73	3.66	9147	17225	**26372**	1.15	0.55	**0.75**
95-99	20	23	**43**	0.37	0.42	0.79	2571	5410	**7981**	0.78	0.43	**0.54**
100-104	2	2	**4**	0.04	0.04	0.07	374	665	**1039**	0.53	0.30	**0.38**
105-110	1	0	**1**	0.02	0.00	0.02	87	177	**264**	1.15	0.00	**0.38**
**Total**	**2741**	**2695**	**5436**	**50.42**	**49.58**		**1431396**	**1551517**	**2982913**	**0.19**	**0.17**	**0.18**

eTable 32. Connective tissue cancer, sarcoma prevalence in adults and proportions of patient population by age and sex

	Malignancy of connective tissue/sarcoma (n)			Percent of connective tissue/sarcoma adult cancer patients			Total population (n)			Prevalence (%)		
Age group	Male	Female	Total	Male	Female	Total	Male	Female	Total	Male	Female	Total
20-24	16	7	**23**	1.54	0.67	2.21	132509	143680	**276189**	0.01	0.00	**0.01**
25-29	8	13	**21**	0.77	1.25	2.02	156927	159023	**315950**	0.01	0.01	**0.01**
30-34	17	21	**38**	1.64	2.02	3.66	162004	165350	**327354**	0.01	0.01	**0.01**
35-39	16	15	**31**	1.54	1.44	2.98	156661	158737	**315398**	0.01	0.01	**0.01**
40-44	24	43	**67**	2.31	4.14	6.45	145144	143572	**288716**	0.02	0.03	**0.02**
45-49	19	26	**45**	1.83	2.50	4.33	115926	112939	**228865**	0.02	0.02	**0.02**
50-54	20	28	**48**	1.92	2.69	4.62	93815	98655	**192470**	0.02	0.03	**0.02**
55-59	34	44	**78**	3.27	4.23	7.51	91223	101690	**192913**	0.04	0.04	**0.04**
60-64	40	51	**91**	3.85	4.91	8.76	93150	105570	**198720**	0.04	0.05	**0.05**
65-69	49	60	**109**	4.72	5.77	10.49	93093	106207	**199300**	0.05	0.06	**0.05**
70-74	76	81	**157**	7.31	7.80	15.11	76573	90083	**166656**	0.10	0.09	**0.09**
75-79	62	55	**117**	5.97	5.29	11.26	45011	56829	**101840**	0.14	0.10	**0.11**
80-84	56	50	**106**	5.39	4.81	10.20	36050	51508	**87558**	0.16	0.10	**0.12**
85-89	46	29	**75**	4.43	2.79	7.22	21131	34197	**55328**	0.22	0.08	**0.14**
90-94	12	11	**23**	1.15	1.06	2.21	9147	17225	**26372**	0.13	0.06	**0.09**
95-99	6	4	**10**	0.58	0.38	0.96	2571	5410	**7981**	0.23	0.07	**0.13**
100-104	0	0	**0**	0.00	0.00	0.00	374	665	**1039**	0.00	0.00	**0.00**
105-110	0	0	**0**	0.00	0.00	0.00	87	177	**264**	0.00	0.00	**0.00**
**Total**	**501**	**538**	**1039**	**48.22**	**51.78**		**1431396**	**1551517**	**2982913**	**0.04**	**0.03**	**0.03**

eTable 33. Esophageal cancer prevalence in adults and proportions of patient population by age and sex

	Malignancy of esophagus (n)			Percent of adult esophageal cancer patients			Total population (n)			Prevalence (%)		
**Age group**	**Male**	**Female**	**Total**	**Male**	**Female**	**Total**	**Male**	**Female**	**Total**	**Male**	**Female**	**Total**
20-24	0	0	**0**	0.00	0.00	0.00	132509	143680	**276189**	0.00	0.00	**0.00**
25-29	1	1	**2**	0.38	0.38	0.75	156927	159023	**315950**	0.00	0.00	**0.00**
30-34	2	0	**2**	0.75	0.00	0.75	162004	165350	**327354**	0.00	0.00	**0.00**
35-39	2	4	**6**	0.75	1.50	2.26	156661	158737	**315398**	0.00	0.00	**0.00**
40-44	4	4	**8**	1.50	1.50	3.01	145144	143572	**288716**	0.00	0.00	**0.00**
45-49	5	2	**7**	1.88	0.75	2.63	115926	112939	**228865**	0.00	0.00	**0.00**
50-54	6	5	**11**	2.26	1.88	4.14	93815	98655	**192470**	0.01	0.01	**0.01**
55-59	16	3	**19**	6.02	1.13	7.14	91223	101690	**192913**	0.02	0.00	**0.01**
60-64	21	12	**33**	7.89	4.51	12.41	93150	105570	**198720**	0.02	0.01	**0.02**
65-69	28	15	**43**	10.53	5.64	16.17	93093	106207	**199300**	0.03	0.01	**0.02**
70-74	20	17	**37**	7.52	6.39	13.91	76573	90083	**166656**	0.03	0.02	**0.02**
75-79	19	16	**35**	7.14	6.02	13.16	45011	56829	**101840**	0.04	0.03	**0.03**
80-84	17	14	**31**	6.39	5.26	11.65	36050	51508	**87558**	0.05	0.03	**0.04**
85-89	10	10	**20**	3.76	3.76	7.52	21131	34197	**55328**	0.05	0.03	**0.04**
90-94	5	5	**10**	1.88	1.88	3.76	9147	17225	**26372**	0.05	0.03	**0.04**
95-99	0	2	**2**	0.00	0.75	0.75	2571	5410	**7981**	0.00	0.04	**0.03**
100-104	0	0	**0**	0.00	0.00	0.00	374	665	**1039**	0.00	0.00	**0.00**
105-110	0	0	**0**	0.00	0.00	0.00	87	177	**264**	0.00	0.00	**0.00**
**Total**	**156**	**110**	**266**	**58.65**	**41.35**		**1431396**	**1551517**	**2982913**	**0.01**	**0.01**	**0.01**

eTable 34. Kidney cancer prevalence in adults and proportions of patient population by age and sex

	Malignancy of kidney (n)			Percent of adult kidney cancer patients			Total population (n)			Prevalence (%)		
**Age group**	**Male**	**Female**	**Total**	**Male**	**Female**	**Total**	**Male**	**Female**	**Total**	**Male**	**Female**	**Total**
20-24	1	1	**2**	0.05	0.05	0.10	132509	143680	**276189**	0.00	0.00	**0.00**
25-29	3	5	**8**	0.14	0.24	0.39	156927	159023	**315950**	0.00	0.00	**0.00**
30-34	13	11	**24**	0.63	0.53	1.16	162004	165350	**327354**	0.01	0.01	**0.01**
35-39	18	8	**26**	0.87	0.39	1.25	156661	158737	**315398**	0.01	0.01	**0.01**
40-44	28	23	**51**	1.35	1.11	2.46	145144	143572	**288716**	0.02	0.02	**0.02**
45-49	44	22	**66**	2.12	1.06	3.19	115926	112939	**228865**	0.04	0.02	**0.03**
50-54	55	31	**86**	2.65	1.50	4.15	93815	98655	**192470**	0.06	0.03	**0.04**
55-59	91	40	**131**	4.39	1.93	6.32	91223	101690	**192913**	0.10	0.04	**0.07**
60-64	149	61	**210**	7.19	2.94	10.14	93150	105570	**198720**	0.16	0.06	**0.11**
65-69	240	122	**362**	11.58	5.89	17.47	93093	106207	**199300**	0.26	0.11	**0.18**
70-74	265	142	**407**	12.79	6.85	19.64	76573	90083	**166656**	0.35	0.16	**0.24**
75-79	151	88	**239**	7.29	4.25	11.53	45011	56829	**101840**	0.34	0.15	**0.23**
80-84	153	85	**238**	7.38	4.10	11.49	36050	51508	**87558**	0.42	0.17	**0.27**
85-89	98	52	**150**	4.73	2.51	7.24	21131	34197	**55328**	0.46	0.15	**0.27**
90-94	38	19	**57**	1.83	0.92	2.75	9147	17225	**26372**	0.42	0.11	**0.22**
95-99	8	6	**14**	0.39	0.29	0.68	2571	5410	**7981**	0.31	0.11	**0.18**
100-104	1	0	**1**	0.05	0.00	0.05	374	665	**1039**	0.27	0.00	**0.10**
105-110	0	0	**0**	0.00	0.00	0.00	87	177	**264**	0.00	0.00	**0.00**
**Total**	**1356**	**716**	**2072**	**65.44**	**34.56**		**1431396**	**1551517**	**2982913**	**0.09**	**0.05**	**0.07**

eTable 35. Laryngeal cancer prevalence in adults and proportions of patient population by age and sex

	Malignancy of larynx (n)			Percent of adult laryngeal cancer patients			Total population (n)			Prevalence (%)		
**Age group**	**Male**	**Female**	**Total**	**Male**	**Female**	**Total**	**Male**	**Female**	**Total**	**Male**	**Female**	**Total**
20-24	1	1	**2**	0.19	0.19	0.38	132509	143680	**276189**	0.00	0.00	**0.00**
25-29	0	3	**3**	0.00	0.57	0.57	156927	159023	**315950**	0.00	0.00	**0.00**
30-34	1	1	**2**	0.19	0.19	0.38	162004	165350	**327354**	0.00	0.00	**0.00**
35-39	5	2	**7**	0.95	0.38	1.33	156661	158737	**315398**	0.00	0.00	**0.00**
40-44	8	3	**11**	1.52	0.57	2.09	145144	143572	**288716**	0.01	0.00	**0.00**
45-49	7	9	**16**	1.33	1.71	3.04	115926	112939	**228865**	0.01	0.01	**0.01**
50-54	20	10	**30**	3.80	1.90	5.70	93815	98655	**192470**	0.02	0.01	**0.02**
55-59	41	18	**59**	7.79	3.42	11.22	91223	101690	**192913**	0.04	0.02	**0.03**
60-64	48	18	**66**	9.13	3.42	12.55	93150	105570	**198720**	0.05	0.02	**0.03**
65-69	74	25	**99**	14.07	4.75	18.82	93093	106207	**199300**	0.08	0.02	**0.05**
70-74	57	20	**77**	10.84	3.80	14.64	76573	90083	**166656**	0.07	0.02	**0.05**
75-79	41	12	**53**	7.79	2.28	10.08	45011	56829	**101840**	0.09	0.02	**0.05**
80-84	36	16	**52**	6.84	3.04	9.89	36050	51508	**87558**	0.10	0.03	**0.06**
85-89	24	5	**29**	4.56	0.95	5.51	21131	34197	**55328**	0.11	0.01	**0.05**
90-94	11	2	**13**	2.09	0.38	2.47	9147	17225	**26372**	0.12	0.01	**0.05**
95-99	6	1	**7**	1.14	0.19	1.33	2571	5410	**7981**	0.23	0.02	**0.09**
100-104	0	0	**0**	0.00	0.00	0.00	374	665	**1039**	0.00	0.00	**0.00**
105-110	0	0	**0**	0.00	0.00	0.00	87	177	**264**	0.00	0.00	**0.00**
**Total**	**380**	**146**	**526**	**72.24**	**27.76**		**1431396**	**1551517**	**2982913**	**0.03**	**0.01**	**0.02**

eTable 36. Liver/bile duct cancer prevalence in adults and proportions of patient population by age and sex

	Malignancy of liver/bile ducts (n)			Percent of adult liver/bile duct cancer patients			Total population (n)			Prevalence (%)		
**Age group**	**Male**	**Female**	**Total**	**Male**	**Female**	**Total**	**Male**	**Female**	**Total**	**Male**	**Female**	**Total**
20-24	3	2	**5**	0.53	0.35	0.88	132509	143680	**276189**	0.00	0.00	**0.00**
25-29	0	1	**1**	0.00	0.18	0.18	156927	159023	**315950**	0.00	0.00	**0.00**
30-34	0	5	**5**	0.00	0.88	0.88	162004	165350	**327354**	0.00	0.00	**0.00**
35-39	2	5	**7**	0.35	0.88	1.23	156661	158737	**315398**	0.00	0.00	**0.00**
40-44	6	7	**13**	1.06	1.23	2.29	145144	143572	**288716**	0.00	0.00	**0.00**
45-49	6	6	**12**	1.06	1.06	2.11	115926	112939	**228865**	0.01	0.01	**0.01**
50-54	18	13	**31**	3.17	2.29	5.46	93815	98655	**192470**	0.02	0.01	**0.02**
55-59	21	20	**41**	3.70	3.52	7.22	91223	101690	**192913**	0.02	0.02	**0.02**
60-64	38	22	**60**	6.69	3.87	10.56	93150	105570	**198720**	0.04	0.02	**0.03**
65-69	67	33	**100**	11.80	5.81	17.61	93093	106207	**199300**	0.07	0.03	**0.05**
70-74	58	47	**105**	10.21	8.27	18.49	76573	90083	**166656**	0.08	0.05	**0.06**
75-79	34	41	**75**	5.99	7.22	13.20	45011	56829	**101840**	0.08	0.07	**0.07**
80-84	34	30	**64**	5.99	5.28	11.27	36050	51508	**87558**	0.09	0.06	**0.07**
85-89	18	18	**36**	3.17	3.17	6.34	21131	34197	**55328**	0.09	0.05	**0.07**
90-94	6	5	**11**	1.06	0.88	1.94	9147	17225	**26372**	0.07	0.03	**0.04**
95-99	0	1	**1**	0.00	0.18	0.18	2571	5410	**7981**	0.00	0.02	**0.01**
100-104	0	1	**1**	0.00	0.18	0.18	374	665	**1039**	0.00	0.15	**0.10**
105-110	0	0	**0**	0.00	0.00	0.00	87	177	**264**	0.00	0.00	**0.00**
**Total**	**311**	**257**	**568**	**54.75**	**45.25**		**1431396**	**1551517**	**2982913**	**0.02**	**0.02**	**0.02**

eTable 37. Lung cancer prevalence in adults and proportions of patient population by age and sex

	Malignancy of lung (n)			Percent of adult lung cancer patients			Total Population (n)			Prevalence (%)		
**Age group**	**Male**	**Female**	**Total**	**Male**	**Female**	**Total**	**Male**	**Female**	**Total**	**Male**	**Female**	**Total**
20-24	0	2	**2**	0.00	0.06	0.06	132509	143680	**276189**	0.00	0.00	**0.00**
25-29	1	2	**3**	0.03	0.06	0.09	156927	159023	**315950**	0.00	0.00	**0.00**
30-34	1	6	**7**	0.03	0.17	0.20	162004	165350	**327354**	0.00	0.00	**0.00**
35-39	9	15	**24**	0.26	0.44	0.70	156661	158737	**315398**	0.01	0.01	**0.01**
40-44	21	22	**43**	0.61	0.64	1.25	145144	143572	**288716**	0.01	0.02	**0.01**
45-49	41	26	**67**	1.19	0.76	1.95	115926	112939	**228865**	0.04	0.02	**0.03**
50-54	62	63	**125**	1.80	1.83	3.63	93815	98655	**192470**	0.07	0.06	**0.06**
55-59	158	117	**275**	4.59	3.40	7.99	91223	101690	**192913**	0.17	0.12	**0.14**
60-64	280	182	**462**	8.14	5.29	13.43	93150	105570	**198720**	0.30	0.17	**0.23**
65-69	321	272	**593**	9.33	7.91	17.24	93093	106207	**199300**	0.34	0.26	**0.30**
70-74	398	322	**720**	11.57	9.36	20.93	76573	90083	**166656**	0.52	0.36	**0.43**
75-79	242	221	**463**	7.03	6.42	13.46	45011	56829	**101840**	0.54	0.39	**0.45**
80-84	207	181	**388**	6.02	5.26	11.28	36050	51508	**87558**	0.57	0.35	**0.44**
85-89	106	97	**203**	3.08	2.82	5.90	21131	34197	**55328**	0.50	0.28	**0.37**
90-94	27	29	**56**	0.78	0.84	1.63	9147	17225	**26372**	0.30	0.17	**0.21**
95-99	7	1	**8**	0.20	0.03	0.23	2571	5410	**7981**	0.27	0.02	**0.10**
100-104	0	1	**1**	0.00	0.03	0.03	374	665	**1039**	0.00	0.15	**0.10**
105-110	0	0	**0**	0.00	0.00	0.00	87	177	**264**	0.00	0.00	**0.00**
**Total**	**1881**	**1559**	**3440**	**54.68**	**45.32**		**1431396**	**1551517**	**2982913**	**0.13**	**0.10**	**0.12**

eTable 38. Lymphoma prevalence in adults and proportions of patient population by age and sex

	Lymphoma (n)			Percent of adult lymphoma patients			Total population (n)			Lymphoma prevalence (%)		
**Age group**	**Male**	**Female**	**Total**	**Male**	**Female**	**Total**	**Male**	**Female**	**Total**	**Male**	**Female**	**Total**
20-24	38	38	**76**	0.86	0.86	1.71	132509	143680	**276189**	0.03	0.03	**0.03**
25-29	48	46	**94**	1.08	1.04	2.12	156927	159023	**315950**	0.03	0.03	**0.03**
30-34	62	62	**124**	1.40	1.40	2.80	162004	165350	**327354**	0.04	0.04	**0.04**
35-39	75	83	**158**	1.69	1.87	3.56	156661	158737	**315398**	0.05	0.05	**0.05**
40-44	92	88	**180**	2.08	1.99	4.06	145144	143572	**288716**	0.06	0.06	**0.06**
45-49	81	93	**174**	1.83	2.10	3.93	115926	112939	**228865**	0.07	0.08	**0.08**
50-54	117	118	**235**	2.64	2.66	5.30	93815	98655	**192470**	0.12	0.12	**0.12**
55-59	167	141	**308**	3.77	3.18	6.95	91223	101690	**192913**	0.18	0.14	**0.16**
60-64	219	202	**421**	4.94	4.56	9.50	93150	105570	**198720**	0.24	0.19	**0.21**
65-69	337	324	**661**	7.60	7.31	14.91	93093	106207	**199300**	0.36	0.31	**0.33**
70-74	354	351	**705**	7.99	7.92	15.90	76573	90083	**166656**	0.46	0.39	**0.42**
75-79	269	242	**511**	6.07	5.46	11.53	45011	56829	**101840**	0.60	0.43	**0.50**
80-84	210	232	**442**	4.74	5.23	9.97	36050	51508	**87558**	0.58	0.45	**0.50**
85-89	129	108	**237**	2.91	2.44	5.35	21131	34197	**55328**	0.61	0.32	**0.43**
90-94	40	41	**81**	0.90	0.92	1.83	9147	17225	**26372**	0.44	0.24	**0.31**
95-99	8	16	**24**	0.18	0.36	0.54	2571	5410	**7981**	0.31	0.30	**0.30**
100-104	1	1	**2**	0.02	0.02	0.05	374	665	**1039**	0.27	0.15	**0.19**
105-110	0	0	**0**	0.00	0.00	0.00	87	177	**264**	0.00	0.00	**0.00**
**Total**	**2247**	**2186**	**4433**	**50.69**	**49.31**		**1431396**	**1551517**	**2982913**	**0.16**	**0.14**	**0.15**

eTable 39. Melanoma prevalence in adults and proportions of patient population by age and sex

	Melanoma (n)			Percent of adult melanoma patients			Total Population (n)			Prevalence (%)		
Age group	Male	Female	Total	Male	Female	Total	Male	Female	Total	Male	Female	Total
20-24	1	10	**11**	0.03	0.27	0.29	132509	143680	**276189**	0.00	0.01	**0.00**
25-29	13	19	**32**	0.35	0.51	0.85	156927	159023	**315950**	0.01	0.01	**0.01**
30-34	26	36	**62**	0.69	0.96	1.65	162004	165350	**327354**	0.02	0.02	**0.02**
35-39	38	50	**88**	1.01	1.33	2.34	156661	158737	**315398**	0.02	0.03	**0.03**
40-44	54	80	**134**	1.44	2.13	3.57	145144	143572	**288716**	0.04	0.06	**0.05**
45-49	61	80	**141**	1.62	2.13	3.75	115926	112939	**228865**	0.05	0.07	**0.06**
50-54	69	74	**143**	1.84	1.97	3.81	93815	98655	**192470**	0.07	0.08	**0.07**
55-59	82	108	**190**	2.18	2.87	5.06	91223	101690	**192913**	0.09	0.11	**0.10**
60-64	160	143	**303**	4.26	3.81	8.06	93150	105570	**198720**	0.17	0.14	**0.15**
65-69	275	228	**503**	7.32	6.07	13.38	93093	106207	**199300**	0.30	0.21	**0.25**
70-74	412	308	**720**	10.96	8.20	19.16	76573	90083	**166656**	0.54	0.34	**0.43**
75-79	258	215	**473**	6.87	5.72	12.59	45011	56829	**101840**	0.57	0.38	**0.46**
80-84	299	203	**502**	7.96	5.40	13.36	36050	51508	**87558**	0.83	0.39	**0.57**
85-89	169	111	**280**	4.50	2.95	7.45	21131	34197	**55328**	0.80	0.32	**0.51**
90-94	76	59	**135**	2.02	1.57	3.59	9147	17225	**26372**	0.83	0.34	**0.51**
95-99	24	13	**37**	0.64	0.35	0.98	2571	5410	**7981**	0.93	0.24	**0.46**
100-104	3	1	**4**	0.08	0.03	0.11	374	665	**1039**	0.80	0.15	**0.38**
105-110	0	0	**0**	0.00	0.00	0.00	87	177	**264**	0.00	0.00	**0.00**
**Total**	**2020**	**1738**	**3758**	**53.75**	**46.25**		**1431396**	**1551517**	**2982913**	**0.14**	**0.11**	**0.13**

eTable 40. Multiple myeloma prevalence in adults and proportions of patient population by age and sex

	Multiple myeloma (n)			Percent of adult multiple myeloma patients			Total population (n)			Prevalence (%)		
Age group	Male	Female	Total	Male	Female	Total	Male	Female	Total	Male	Female	Total
20-24	0	1	**1**	0.00	0.06	0.06	132509	143680	**276189**	0.00	0.00	**0.00**
25-29	0	3	**3**	0.00	0.19	0.19	156927	159023	**315950**	0.00	0.00	**0.00**
30-34	3	2	**5**	0.19	0.13	0.32	162004	165350	**327354**	0.00	0.00	**0.00**
35-39	6	5	**11**	0.38	0.32	0.70	156661	158737	**315398**	0.00	0.00	**0.00**
40-44	12	9	**21**	0.77	0.58	1.34	145144	143572	**288716**	0.01	0.01	**0.01**
45-49	18	26	**44**	1.15	1.66	2.82	115926	112939	**228865**	0.02	0.02	**0.02**
50-54	27	28	**55**	1.73	1.79	3.52	93815	98655	**192470**	0.03	0.03	**0.03**
55-59	57	47	**104**	3.65	3.01	6.65	91223	101690	**192913**	0.06	0.05	**0.05**
60-64	103	74	**177**	6.59	4.73	11.32	93150	105570	**198720**	0.11	0.07	**0.09**
65-69	129	110	**239**	8.25	7.04	15.29	93093	106207	**199300**	0.14	0.10	**0.12**
70-74	172	148	**320**	11.00	9.47	20.47	76573	90083	**166656**	0.22	0.16	**0.19**
75-79	125	105	**230**	8.00	6.72	14.72	45011	56829	**101840**	0.28	0.18	**0.23**
80-84	102	106	**208**	6.53	6.78	13.31	36050	51508	**87558**	0.28	0.21	**0.24**
85-89	59	50	**109**	3.77	3.20	6.97	21131	34197	**55328**	0.28	0.15	**0.20**
90-94	18	13	**31**	1.15	0.83	1.98	9147	17225	**26372**	0.20	0.08	**0.12**
95-99	1	3	**4**	0.06	0.19	0.26	2571	5410	**7981**	0.04	0.06	**0.05**
100-104	1	0	**1**	0.06	0.00	0.06	374	665	**1039**	0.27	0.00	**0.10**
105-110	0	0	**0**	0.00	0.00	0.00	87	177	**264**	0.00	0.00	**0.00**
**Total**	**833**	**730**	**1563**	**53.29**	**46.71**		**1431396**	**1551517**	**2982913**	**0.06**	**0.05**	**0.05**

eTable 41. Ovarian cancer prevalence in women and proportions of patient population by age and sex

	Malignancy of ovary (n)	Percent of ovarian cancer patients	Total population (n)	Prevalence (%)
**Age group**	**Female**	**Female**	**Female**	**Female**
20-24	2	0.19	143680	0.00
25-29	12	1.17	159023	0.01
30-34	18	1.75	165350	0.01
35-39	19	1.85	158737	0.01
40-44	52	5.06	143572	0.04
45-49	52	5.06	112939	0.05
50-54	73	7.10	98655	0.07
55-59	80	7.78	101690	0.08
60-64	128	12.45	105570	0.12
65-69	180	17.51	106207	0.17
70-74	181	17.61	90083	0.20
75-79	117	11.38	56829	0.21
80-84	82	7.98	51508	0.16
85-89	24	2.33	34197	0.07
90-94	6	0.58	17225	0.03
95-99	2	0.19	5410	0.04
100-104	0	0.00	665	0.00
105-110	0	0.00	177	0.00
**Total**	**1028**		**1551517**	**0.07**

eTable 42. Pancreatic cancer prevalence in adults and proportions of patient population by age and sex

	Malignancy of pancreas (n)			Percent of adult pancreatic cancer patients			Total population (n)			Prevalence (%)		
**Age group**	**Male**	**Female**	**Total**	**Male**	**Female**	**Total**	**Male**	**Female**	**Total**	**Male**	**Female**	**Total**
20-24	2	2	**4**	0.23	0.23	0.46	132509	143680	**276189**	0.00	0.00	**0.00**
25-29	2	2	**4**	0.23	0.23	0.46	156927	159023	**315950**	0.00	0.00	**0.00**
30-34	2	5	**7**	0.23	0.58	0.81	162004	165350	**327354**	0.00	0.00	**0.00**
35-39	5	6	**11**	0.58	0.70	1.28	156661	158737	**315398**	0.00	0.00	**0.00**
40-44	3	7	**10**	0.35	0.81	1.16	145144	143572	**288716**	0.00	0.00	**0.00**
45-49	6	15	**21**	0.70	1.74	2.44	115926	112939	**228865**	0.01	0.01	**0.01**
50-54	24	23	**47**	2.79	2.67	5.46	93815	98655	**192470**	0.03	0.02	**0.02**
55-59	33	16	**49**	3.83	1.86	5.69	91223	101690	**192913**	0.04	0.02	**0.03**
60-64	57	45	**102**	6.62	5.23	11.85	93150	105570	**198720**	0.06	0.04	**0.05**
65-69	81	76	**157**	9.41	8.83	18.23	93093	106207	**199300**	0.09	0.07	**0.08**
70-74	101	84	**185**	11.73	9.76	21.49	76573	90083	**166656**	0.13	0.09	**0.11**
75-79	52	57	**109**	6.04	6.62	12.66	45011	56829	**101840**	0.12	0.10	**0.11**
80-84	48	45	**93**	5.57	5.23	10.80	36050	51508	**87558**	0.13	0.09	**0.11**
85-89	19	20	**39**	2.21	2.32	4.53	21131	34197	**55328**	0.09	0.06	**0.07**
90-94	5	14	**19**	0.58	1.63	2.21	9147	17225	**26372**	0.05	0.08	**0.07**
95-99	2	1	**3**	0.23	0.12	0.35	2571	5410	**7981**	0.08	0.02	**0.04**
100-104	1	0	**1**	0.12	0.00	0.12	374	665	**1039**	0.27	0.00	**0.10**
105-110	0	0	**0**	0.00	0.00	0.00	87	177	**264**	0.00	0.00	**0.00**
**Total**	**443**	**418**	**861**	**51.45**	**48.55**		**1431396**	**1551517**	**2982913**	**0.03**	**0.03**	**0.03**

eTable 43. Pharyngeal cancer prevalence in adults and proportions of patient population by age and sex

	Malignancy of pharynx (n)			Percent of adult pharyngeal cancer patients			Total population (n)			Prevalence (%)		
**Age group**	**Male**	**Female**	**Total**	**Male**	**Female**	**Total**	**Male**	**Female**	**Total**	**Male**	**Female**	**Total**
20-24	2	2	**4**	0.25	0.25	0.50	132509	143680	**276189**	0.00	0.00	**0.00**
25-29	7	4	**11**	0.87	0.50	1.37	156927	159023	**315950**	0.00	0.00	**0.00**
30-34	6	7	**13**	0.75	0.87	1.62	162004	165350	**327354**	0.00	0.00	**0.00**
35-39	9	3	**12**	1.12	0.37	1.49	156661	158737	**315398**	0.01	0.00	**0.00**
40-44	20	14	**34**	2.49	1.74	4.23	145144	143572	**288716**	0.01	0.01	**0.01**
45-49	18	14	**32**	2.24	1.74	3.99	115926	112939	**228865**	0.02	0.01	**0.01**
50-54	33	19	**52**	4.11	2.37	6.48	93815	98655	**192470**	0.04	0.02	**0.03**
55-59	40	30	**70**	4.98	3.74	8.72	91223	101690	**192913**	0.04	0.03	**0.04**
60-64	61	22	**83**	7.60	2.74	10.34	93150	105570	**198720**	0.07	0.02	**0.04**
65-69	75	44	**119**	9.34	5.48	14.82	93093	106207	**199300**	0.08	0.04	**0.06**
70-74	72	45	**117**	8.97	5.60	14.57	76573	90083	**166656**	0.09	0.05	**0.07**
75-79	51	31	**82**	6.35	3.86	10.21	45011	56829	**101840**	0.11	0.05	**0.08**
80-84	42	35	**77**	5.23	4.36	9.59	36050	51508	**87558**	0.12	0.07	**0.09**
85-89	31	29	**60**	3.86	3.61	7.47	21131	34197	**55328**	0.15	0.08	**0.11**
90-94	18	12	**30**	2.24	1.49	3.74	9147	17225	**26372**	0.20	0.07	**0.11**
95-99	4	2	**6**	0.50	0.25	0.75	2571	5410	**7981**	0.16	0.04	**0.08**
100-104	1	0	**1**	0.12	0.00	0.12	374	665	**1039**	0.27	0.00	**0.10**
105-110	0	0	**0**	0.00	0.00	0.00	87	177	**264**	0.00	0.00	**0.00**
**Total**	**490**	**313**	**803**	**61.02**	**38.98**		**1431396**	**1551517**	**2982913**	**0.03**	**0.02**	**0.03**

eTable 44. Prostate cancer prevalence in men, and proportions of patient population by age and sex

	Malignancy of prostate (n)	Percent of prostate cancer patients	Total population (n)	Prevalence (%)
Age group	Male	Male	Male	Male
20-24	0	0.00	132509	0.00
25-29	2	0.03	156927	0.00
30-34	0	0.00	162004	0.00
35-39	1	0.01	156661	0.00
40-44	1	0.01	145144	0.00
45-49	8	0.12	115926	0.01
50-54	65	0.95	93815	0.07
55-59	203	2.95	91223	0.22
60-64	501	7.29	93150	0.54
65-69	1108	16.13	93093	1.19
70-74	1546	22.50	76573	2.02
75-79	1194	17.38	45011	2.65
80-84	1122	16.33	36050	3.11
85-89	710	10.33	21131	3.36
90-94	326	4.75	9147	3.56
95-99	71	1.03	2571	2.76
100-104	12	0.17	374	3.21
105-110	0	0.00	87	0.00
**Total**	**6870**		**1431396**	0.48

eTable 45. Stomach cancer prevalence in adults and proportions of patient population by age and sex

	Stomach Cancer (n)			Percent of adult stomach cancer patients			Total Population (n)			Prevalence (%)		
**Age group**	**Male**	**Female**	**Total**	**Male**	**Female**	**Total**	**Male**	**Female**	**Total**	**Male**	**Female**	**Total**
20-24	1	0	**1**	0.10	0.00	0.10	132509	143680	**276189**	0.00	0.00	**0.00**
25-29	1	3	**4**	0.10	0.31	0.41	156927	159023	**315950**	0.00	0.00	**0.00**
30-34	2	6	**8**	0.21	0.62	0.82	162004	165350	**327354**	0.00	0.00	**0.00**
35-39	4	3	**7**	0.41	0.31	0.72	156661	158737	**315398**	0.00	0.00	**0.00**
40-44	10	13	**23**	1.03	1.33	2.36	145144	143572	**288716**	0.01	0.01	**0.01**
45-49	20	8	**28**	2.05	0.82	2.87	115926	112939	**228865**	0.02	0.01	**0.01**
50-54	21	21	**42**	2.15	2.15	4.31	93815	98655	**192470**	0.02	0.02	**0.02**
55-59	51	27	**78**	5.23	2.77	8.00	91223	101690	**192913**	0.06	0.03	**0.04**
60-64	65	47	**112**	6.67	4.82	11.49	93150	105570	**198720**	0.07	0.04	**0.06**
65-69	77	56	**133**	7.90	5.74	13.64	93093	106207	**199300**	0.08	0.05	**0.07**
70-74	120	77	**197**	12.31	7.90	20.21	76573	90083	**166656**	0.16	0.09	**0.12**
75-79	86	41	**127**	8.82	4.21	13.03	45011	56829	**101840**	0.19	0.07	**0.12**
80-84	68	45	**113**	6.97	4.62	11.59	36050	51508	**87558**	0.19	0.09	**0.13**
85-89	46	31	**77**	4.72	3.18	7.90	21131	34197	**55328**	0.22	0.09	**0.14**
90-94	14	8	**22**	1.44	0.82	2.26	9147	17225	**26372**	0.15	0.05	**0.08**
95-99	1	1	**2**	0.10	0.10	0.21	2571	5410	**7981**	0.04	0.02	**0.03**
100-104	0	0	**0**	0.00	0.00	0.00	374	665	**1039**	0.00	0.00	**0.00**
105-110	1	0	**1**	0.10	0.00	0.10	87	177	**264**	1.15	0.00	**0.38**
**Total**	**588**	**387**	**975**	**60.31**	**39.69**		**1431396**	**1551517**	**2982913**	**0.04**	**0.02**	**0.03**

eTable 46. Thyroid cancer prevalence in adults and proportions of patient population by age and sex

	Malignancy of thyroid (n)			Percent of adult thyroid cancer patients			Total population (n)			Prevalence (%)		
Age group	Male	Female	Total	Male	Female	Total	Male	Female	Total	Male	Female	Total
20-24	6	26	**32**	0.33	1.44	1.77	132509	143680	**276189**	0.00	0.02	**0.01**
25-29	13	45	**58**	0.72	2.49	3.21	156927	159023	**315950**	0.01	0.03	**0.02**
30-34	21	81	**102**	1.16	4.48	5.64	162004	165350	**327354**	0.01	0.05	**0.03**
35-39	26	85	**111**	1.44	4.70	6.14	156661	158737	**315398**	0.02	0.05	**0.04**
40-44	30	123	**153**	1.66	6.80	8.46	145144	143572	**288716**	0.02	0.09	**0.05**
45-49	26	99	**125**	1.44	5.47	6.91	115926	112939	**228865**	0.02	0.09	**0.05**
50-54	32	100	**132**	1.77	5.53	7.30	93815	98655	**192470**	0.03	0.10	**0.07**
55-59	31	117	**148**	1.71	6.47	8.18	91223	101690	**192913**	0.03	0.12	**0.08**
60-64	35	132	**167**	1.93	7.30	9.23	93150	105570	**198720**	0.04	0.13	**0.08**
65-69	61	162	**223**	3.37	8.96	12.33	93093	106207	**199300**	0.07	0.15	**0.11**
70-74	63	183	**246**	3.48	10.12	13.60	76573	90083	**166656**	0.08	0.20	**0.15**
75-79	28	106	**134**	1.55	5.86	7.41	45011	56829	**101840**	0.06	0.19	**0.13**
80-84	33	77	**110**	1.82	4.26	6.08	36050	51508	**87558**	0.09	0.15	**0.13**
85-89	21	30	**51**	1.16	1.66	2.82	21131	34197	**55328**	0.10	0.09	**0.09**
90-94	6	8	**14**	0.33	0.44	0.77	9147	17225	**26372**	0.07	0.05	**0.05**
95-99	1	2	**3**	0.06	0.11	0.17	2571	5410	**7981**	0.04	0.04	**0.04**
100-104	0	0	**0**	0.00	0.00	0.00	374	665	**1039**	0.00	0.00	**0.00**
105-110	0	0	**0**	0.00	0.00	0.00	87	177	**264**	0.00	0.00	**0.00**
**Total**	**433**	**1376**	**1809**	**23.94**	**76.06**		**1431396**	**1551517**	**2982913**	**0.03**	**0.09**	**0.06**

eTable 47. Uterine, cervix cancer prevalence in women, and proportions of patient population by age and sex

	Malignancy of uterus, cervix (n)	Percent of uterine, cervix cancer patients	Total population (n)	Prevalence (%)
**Age group**	**Female**	**Female**	**Female**	**Female**
20-24	24	0.67	143680	0.02
25-29	143	3.97	159023	0.09
30-34	262	7.28	165350	0.16
35-39	338	9.39	158737	0.21
40-44	353	9.80	143572	0.25
45-49	218	6.05	112939	0.19
50-54	191	5.30	98655	0.19
55-59	254	7.05	101690	0.25
60-64	353	9.80	105570	0.33
65-69	427	11.86	106207	0.40
70-74	445	12.36	90083	0.49
75-79	252	7.00	56829	0.44
80-84	190	5.28	51508	0.37
85-89	102	2.83	34197	0.30
90-94	40	1.11	17225	0.23
95-99	7	0.19	5410	0.13
100-104	2	0.06	665	0.30
105-110	0	0.00	177	0.00
**Total**	**3601**		**1551517**	0.23
